# Body mass index versus surrogate measures of central adiposity as independent predictors of mortality in type 2 diabetes

**DOI:** 10.1186/s12933-022-01706-2

**Published:** 2022-12-02

**Authors:** Emanuela Orsi, Anna Solini, Giuseppe Penno, Enzo Bonora, Cecilia Fondelli, Roberto Trevisan, Monica Vedovato, Franco Cavalot, Olga Lamacchia, Jonida Haxhi, Antonio Nicolucci, Giuseppe Pugliese, for the Renal Insufficiency And Cardiovascular Events (RIACE) Study Group, Luigi Laviola, Lucilla Bollanti, Elena Alessi, Martina Vitale, Tiziana Cirrito, Paolo Cavallo-Perin, Gabriella Gruden, Bartolomeo Lorenzati, Mariella Trovati, Leonardo Di Martino, Fabio Mazzaglia, Giampaolo Zerbini, Valentina Martina, Silvia Maestroni, Valentina Capuano, Eva Palmieri, Elena Lunati, Valeria Grancini, Veronica Resi, Antonio Pontiroli, Annamaria Veronelli, Barbara Zecchini, Maura Arosio, Laura Montefusco, Antonio Rossi, Guido Adda, Anna Corsi, Mascia Albizzi, Giacomo Zoppini, Angelo Avogaro, Laura Pucci, Daniela Lucchesi, Eleonora Russo, Monia Garofolo, Francesco Dotta, Laura Nigi, Susanna Morano, Tiziana Filardi, Irene Turinese, Marco Rossetti, Raffaella Buzzetti, Chiara Foffi, Mauro Cignarelli, Sabina Pinnelli, Lucia Monaco, Francesco Giorgino, Annalisa Natalicchio, Giorgio Sesti, Francesco Andreozzi, Marco Giorgio Baroni, Giuseppina Frau, Alessandra Boi

**Affiliations:** 1 Diabetes Unit, Foundation IRCCS Cà Granda Ospedale Maggiore Policlinico, Milan, Italy; 2 Department of Surgical, Medical, Molecular and Critical Area Pathology, University of Pisa, Pisa, Italy; 3 Department of Clinical and Experimental Medicine, University of Pisa, Pisa, Italy; 4 Division of Endocrinology, Diabetes and Metabolism, University and Hospital Trust of Verona, Verona, Italy; 5 Diabetes Unit, University of Siena, Siena, Italy; 6 Endocrinology and Diabetes Unit, Azienda Ospedaliera Papa Giovanni XXIII, Bergamo, Italy; 7 Department of Clinical and Experimental Medicine, University of Padua, Padua, Italy; 8 Department of Clinical and Biological Sciences, University of Turin, Orbassano, Italy; 9 Department of Medical Sciences, University of Foggia, Foggia, Italy; 10 Department of Clinical and Molecular Medicine, “La Sapienza” University, Via Di Grottarossa, 1035-1039, 00189 Rome, Italy; 11 Center for Outcomes Research and Clinical Epidemiology (CORESEARCH), Pescara, Italy

**Keywords:** Type 2 diabetes, Body mass index, Central adiposity, All-cause-mortality, Physical activity

## Abstract

**Background:**

An “obesity paradox” for mortality has been shown in chronic disorders such as diabetes, and attributed to methodological bias, including the use of body mass index (BMI) for obesity definition. This analysis investigated the independent association of BMI versus surrogate measures of central adiposity with all-cause mortality in individuals with type 2 diabetes.

**Methods:**

The Renal Insufficiency And Cardiovascular Events Italian Multicentre Study is a prospective cohort study that enrolled 15,773 patients in 19 Italian centres in 2006–2008. Exposures were BMI and the surrogate measures of central adiposity waist circumference (WC), waist-to-height ratio (WHtR), and A Body Shape Index (ABSI). Vital status was retrieved on 31 October 2015 for 15,656 patients (99.3%),

**Results:**

Age- and sex-adjusted hazard ratios and 95% confidence intervals were significantly higher in BMI-based underweight (1.729 [1.193–2.505), *P* = 0.004), moderately obese (1.214 [1.058–1.392), *P* = 0.006) and severely obese (1.703 [1.402–2.068), *P* < 0.0001), lower in overweight (0.842 [0.775–0.915), *P* < 0.0001) and similar in mildly obese (0.950 [0.864–1.045), *P* = 0.292), compared to normal-weight individuals. When further adjusting for smoking, physical activity (PA), and comorbidities, risk was lower also in mildly obese versus normal-weight patients. The BMI-mortality relationship did not change after sequentially excluding ever smokers, individuals with comorbidities, and those died within two years from enrollment and when analyzing separately participants below and above the median age. Conversely, a paradox relationship was observed among inactive/moderately inactive, but not moderately/highly active patients. Mortality risk adjusted for age, gender, smoking, PA and comorbidities was significantly higher in the highest tertile of WC (1.279 [1.089–1.501], *P* = 0.003), WHtR (1.372 [1.165–1.615], *P* < 0.0001), and ABSI (1.263 [1.067–1.495], *P* = 0.007) versus the lowest tertile. However, risk was lower in the intermediate versus lowest tertile for WC (0.823 [0.693–0.979], *P* = 0.028), similar for WHtR, and higher, though not significantly, for ABSI.

**Conclusions:**

An “overweight paradox” remained after controlling for age, smoking, and comorbidities, arguing against a collider bias or reverse causation. However, it could be partly explained by confounding from PA level, possibly through its impact on lean mass and cardiorespiratory fitness. No obesity paradox was observed with WHtR and especially ABSI, which predicted mortality risk associated with central adiposity better than WC.

*Trial registration* ClinicalTrials.gov, NCT00715481, 15 July, 2008

**Supplementary Information:**

The online version contains supplementary material available at 10.1186/s12933-022-01706-2.

## Background

In the general population, higher body mass index (BMI) is associated with increased all-cause mortality [1], with the nadir of the curve generally found in the upper normal-weight range [2], though the relationship is J-shaped [3], as also underweight carries an increased risk of death. However, numerous epidemiological surveys have reported an association of increased BMI with decreased mortality in older individuals as well as in patients in acute clinical settings or suffering from several chronic disorders, suggesting that a mild-to-moderate excess of fat might be protective under these conditions [4].

Indeed, this so-called “obesity paradox” has been attributed to misclassification bias caused by methodological problems. Potential sources of bias include residual or unmeasured confounding, if relevant variables are not taken into account. An example is physical fitness, including cardiorespiratory and muscle fitness, which are both associated with better survival irrespective of BMI [5]. An inverse relationship between exposure (obesity) and outcome (death) has also been related to reverse causation, as unintentional weight loss may be a consequence of several, potentially fatal illnesses, thereby increasing mortality among previously overweight or obese individuals who had become non-obese because of the disease [6]. Another reason can be selection bias, including survivor bias and collider bias. A survivor bias may occur if the most obese and sickest individuals have already died at the time of enrolment, though obese patients might also have better outcomes because they are treated more aggressively than non-obese individuals [7]. A collider bias may be due to smoking, which is inversely related to BMI and is a stronger risk factor for mortality than obesity itself, thus potentially reversing the direction of the association between the two [8].

However, the main source of bias might be the use of BMI as a measure of obesity, as weight reflects not only fat mass but also lean (muscle) mass and does not provide information about the central (visceral) versus peripheral (subcutaneous) distribution of fat accumulation [9]. Hence, differences in the relative contributions to BMI of “harmful” fat mass and central fat versus “protective” lean mass and peripheral fat have been claimed for explaining the obesity paradox [9]. The reported weakening of the BMI-mortality relationship with increasing age [10] may in fact be related to the combination of decreased muscle mass and increased central fat in the context of an overall reduction of body weight (and BMI) characterizing older individuals [7]. Consistently, surrogate measures of visceral adiposity, such as waist circumference (WC) and waist-to-hip ratio (WHR), were reported to be linearly related to death in subjects with coronary artery disease [11] and recent evidence indicates that two WC-derived measures, waist-to-height ratio (WHtR) [12] and A Body Shape Index (ABSI) [13] predict mortality even better than WC and WHR.

An obesity paradox has been almost consistently shown in people with type 2 diabetes [14, 15], with the lowest mortality in those with a BMI in the overweight [16–19] or even the obesity [20–24] range. However, in these individuals, the BMI-mortality relationship may be strongly influenced by confounding due to reverse causation from associated comorbidities or low fitness and related changes in body composition [25, 26]. Two studies using a multiple obesity index approach have in fact shown that either WHtR [27] or ABSI [28] are superior to both BMI and WC as predictors of mortality also in patients with type 2 diabetes. In order to confirm and extend these observations, the present analysis aimed at assessing the independent association of BMI versus surrogate measures of central adiposity (including both WHtR and ABSI) with death from any cause in the large cohort of well-characterized individuals with type 2 diabetes from the Renal Insufficiency And Cardiovascular Events (RIACE) Italian Multicentre Study, which allows accounting for several potential sources of bias.

## Methods

### Design

The RIACE is an observational, prospective, cohort study on the impact of estimated glomerular filtration rate (eGFR) on morbidity and mortality in individuals with type 2 diabetes [29]. The study was conducted in accordance with the Declaration of Helsinki. The research protocol was approved by the locally appointed ethics committees and participants provided an informed consent.

### Patients

The study population included 15,773 Caucasian patients (after excluding 160 individuals with missing or implausible values), consecutively attending 19 hospital-based, tertiary referral Diabetes Clinics of the National Health Service throughout Italy in the years 2006–2008. Exclusion criteria were dialysis or renal transplantation.

### All-cause mortality

The vital status of study participants on 31 October 2015 was verified by interrogating the Italian Health Card database (http://sistemats1.sanita.finanze.it/wps/portal/), which provides updated and reliable information on all current Italian residents [30].

### Baseline measurements

Baseline data were collected using a standardized protocol across participating centres [29].

Participants underwent a structured interview in order to collect the following information: age at the time of the interview, smoking status, physical activity (PA) level; known diabetes duration, current glucose-, lipid-, and blood pressure (BP)-lowering treatments, and severe comorbidities. Patients were categorized by smoking status as never, former, or current smokers and by moderate-to-vigorous PA level as physically inactive or moderately inactive (< 60 min·week^−1^), moderately active (60–150 min·week^−1^), or highly active (> 150 min·week^−1^). Comorbidities included chronic obstructive pulmonary disease (COPD), chronic liver disease, and cancer.

The BMI was calculated from weight and height and BP was measured with a sphygmomanometer with the patients seated with the arm at the heart level. Moreover, WC was measured at the umbilicus and then divided by height to obtain WHtR [12, 27] and used together with BMI and height for calculating ABSI by the formula: WC/(BMI^2/3^ × height^1/2^) [13, 28].

Haemoglobin A_1c_ (HbA_1c_) and fasting levels of triglycerides and total and HDL cholesterol were measured by standard methods. The triglyceride:HDL cholesterol ratio (TG:HDL) was then calculated by dividing triglyceride for HDL cholesterol levels (both in mg/dl) and LDL cholesterol was estimated by the Friedewald formula.

The presence of diabetic kidney disease (DKD) was assessed by measuring albuminuria and serum creatinine, as previously detailed [31]. Patients were then assigned to one of the following DKD phenotypes: no DKD, albuminuria alone (albuminuric DKD with preserved eGFR), reduced eGFR alone (non-albuminuric DKD), or both albuminuria and reduced eGFR (albuminuric DKD with reduced eGFR).

In each centre, the presence of diabetic retinopathy (DR) was assessed by an expert ophthalmologist by dilated fundoscopy. Patients with mild or moderate non-proliferative DR were classified as having non-advanced DR, whereas those with severe non-proliferative DR, proliferative DR, or maculopathy were grouped into the advanced DR category. DR grade was assigned based on the worse eye [32].

Previous major acute CVD events, including myocardial infarction; stroke; foot ulcer/gangrene/amputation; and coronary, carotid, and lower limb revascularization, were adjudicated based on hospital discharge records by an ad hoc committee in each centre [29].

The above data were obtained from all participants, except for WC, which was available only from 5 out 19 centres (4618 individuals), and LDL cholesterol, which was calculable only for 15,501 patients because of triglyceride levels exceeding 4.5 mmol l^−1^ in the remaining 272 individuals.

### Statistical analysis

For the purpose of the current analysis, the RIACE cohort was divided into the following BMI categories (kg·m^−2^): underweight (< 18.5), normal-weight (18.5–24.9), overweight (25.0–29.9), grade I or mild obesity (30.0–34.9), grade II or moderate obesity (35.0–39.9), and grade III or severe obesity (≥ 40.0). In addition, the individuals with available WC measurements were divided into sex-specific tertiles of WC, WHtR, and ABSI, three surrogate measures of central adiposity.

Data are expressed as mean ± SD or median (interquartile range) for continuous variables, and number of cases and percentage for categorical variables. Comparisons among groups were performed by one-way ANOVA for continuous variables and by Pearson’s χ^2^ test for categorical variables.

Crude mortality rates were described as events per 1000 patient-years, with 95% exact Poisson confidence intervals (CIs) and adjusted for age and sex by a Poisson regression model. Kaplan–Meier survival probabilities for all-cause mortality were estimated according to BMI categories and WC or WHtR tertiles and differences were analysed using the log-rank statistic. The hazard ratios (HRs) and their 95% CIs were estimated by Cox proportional hazards regression, using the normal-weight category as reference. These analyses were adjusted for age and sex (model 1), plus smoking, PA level, and severe comorbidities (model 2), plus CVD risk factors, i.e., diabetes duration, HbA_1c_, triglycerides, total and HDL cholesterol, and systolic and diastolic BP, and treatment, i.e., anti-hyperglycaemic, lipid-lowering, and anti-hypertensive therapy (model 3), and plus presence of complications, i.e., DKD phenotypes, DR grade and any CVD (model 4). The analyses by WC and WHtR tertiles were adjusted also for BMI. All the analyses were repeated separately for men and women. In addition, the analyses by BMI categories were repeated (a) separately in participants below and above the median age (i.e., 67.25 years) and in physically inactive or moderately inactive versus moderately and highly active individuals; and (b) after sequentially excluding former or current smokers, patients with comorbidities, and those who died within two years since enrolment.

All *p* values were two-sided, and a *p* < 0.05 was considered statistically significant. Statistical analyses were performed using SPSS version 13.0 (SPSS Inc., Chicago, IL, USA).

## Results

### Overall mortality in the study population

Valid information on vital status was retrieved for 99.3% of participants (15,656 out of 15,773) and 99.1% of those with available WC values (4,578 out of 4,618). At the time of the census, 3,602 (23.0%) individuals had died; death rate was 31.0 per 1000 person-years (95% CI 30.0, 32.0) over a mean follow-up of 7.4 ± 2.1 years, as previously reported [31].

### Clinical features and mortality by BMI categories

The baseline clinical features of the RIACE participants stratified by BMI categories are shown in Additional file 2: Table S1. Age, proportion of current smokers, PA level, diabetes duration, and HDL cholesterol decreased, whereas HbA_1c_, triglycerides, triglycerides:HDL ratio, systolic and diastolic BP, and prevalence of anti-hypertensive treatment increased from the lowest to the highest BMI category. Moreover, a U-shaped trend was observed for proportion of females, albuminuria, eGFR, and prevalence of insulin and anti-coagulant treatment. Finally, underweight individuals showed the lowest prevalence of any CVD, whereas the highest prevalence of chronic liver disease and cancer and the lowest prevalence of COPD were observed in the underweight group and opposite figures were detected in the severely obese group.

**Fig. 1:**
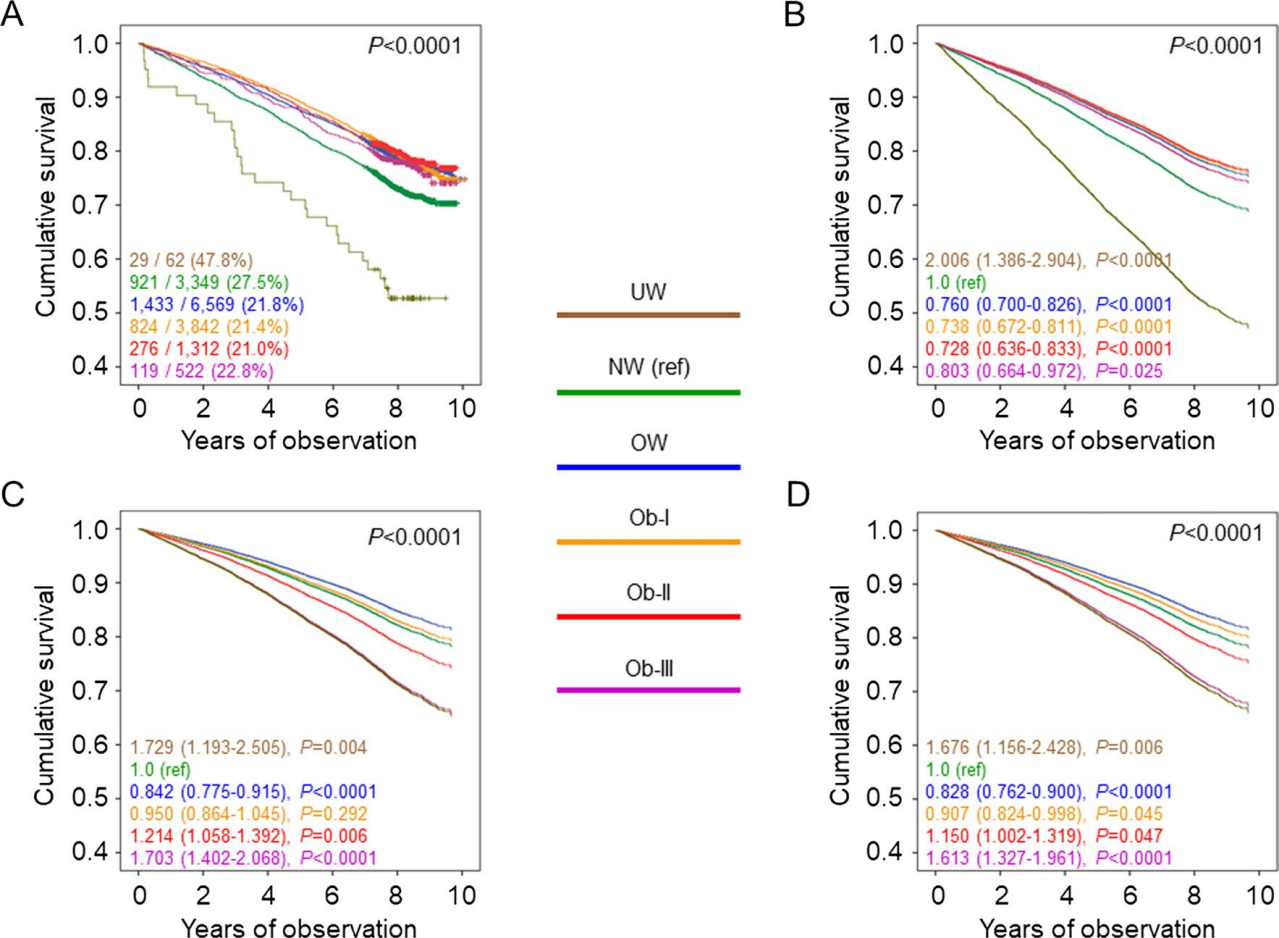
Survival analysis by BMI categories. Kaplan–Meier analysis (**A**) and Cox proportional hazards regression, unadjusted (**B**) and adjusted for age and sex (**C**), and age, sex, smoking status, PA level, and comorbidities (**D**), according to BMI categories. Numbers (percentages) of deaths and HRs (95% CI) for mortality are shown for each group. BMI: body mass index; PA: physical activity; HR: hazard ratio; CI: confidence interval; UW: underweight; NW: normal-weight; OW: overweight; Ob-I: grade I obesity; Ob-II: grade II obesity; Ob-III: grade III obesity

**Fig. 2:**
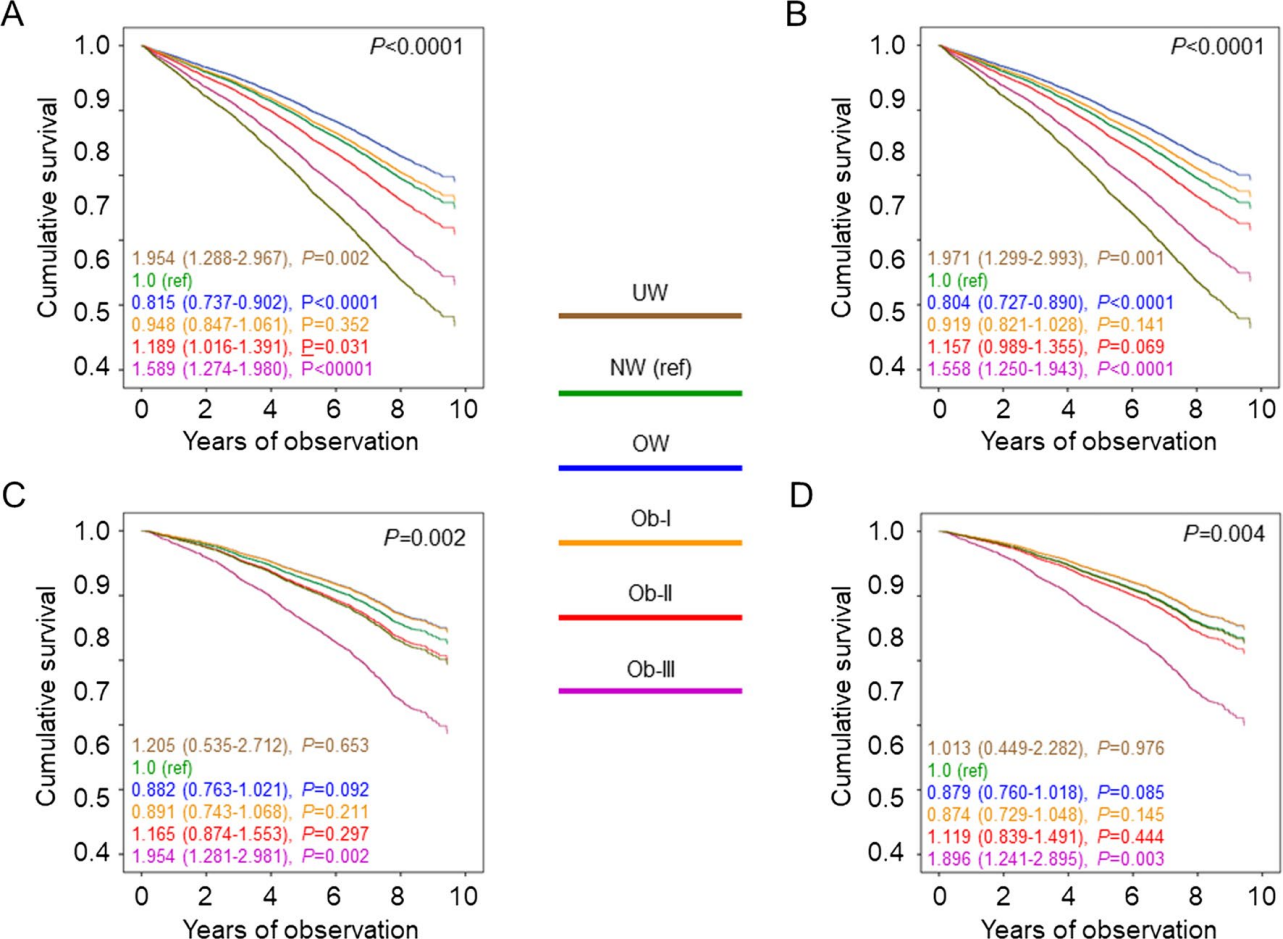
Survival analysis by PA level. Cox proportional hazards regression, adjusted for age and sex (**A**, **C**) and age, sex, smoking status, and comorbidities (**B**, **D**), according to BMI categories, in inactive or moderately inactive (**A**, **B**) and moderately or highly active (**C**, **D**) patients. HRs (95% CI) for mortality are shown for each group. PA: physical activity; BMI: body mass index; HR: hazard ratio; CI: confidence interval; UW: underweight; NW: normal-weight; OW: overweight; Ob-I: grade I obesity; Ob-II: grade II obesity; Ob-III: grade III obesity

Percent deaths (Additional file 2: Table S1), crude mortality rates (Table 1), Kaplan–Meier estimates (Fig. 1A), and unadjusted HRs (Fig. 1B) were higher in underweight and, to a lesser extent, normal-weight participants versus all other BMI categories. When adjusted for age and sex, mortality rates (Table 1) and HRs (Fig. 1C) remained significantly higher in underweight and significantly lower in overweight, as compared with normal-weight individuals; however, mortality became higher in severely obese and, to a lesser extent, moderately obese than in normal-weight participants, as age was much higher in the latter group. When adjusting also for smoking, PA level, and comorbidities, mortality risk was significantly lower also in mildly obese versus normal-weight individuals (Fig. 1D). When further adjusting for CVD risk factors and complications, mortality risk was not significantly different in mildly and moderately obese versus normal weight individuals (not shown). The HRs were similar in patients below (Additional file 3: Fig. S1A, B) and above (Additional file 3: Fig. S1C, D) the median age; conversely, the U-shaped relationship between BMI and mortality was maintained in inactive or moderately inactive participants (Fig. 2A, B), but not in moderately or highly active individuals (Fig. 2C, D). Moreover, when sequentially excluding former and current smokers, participants with comorbidities, and those who died within 2 years from enrolment, mortality risk in overweight and mildly obese patients remained lower and not significantly different, respectively, versus normal-weight individuals (Additional file 4: Fig. S2). Results were similar in males and females (Additional file 5: Table S2).

**Table 1:** Mortality rates in study participants by BMI categories and tertiles of WC, WHtR, and ABSI

	N	Events	Percent events	Events per 1000 patient-years (95% CI), unadjusted	*P*	Events per 1000 patient-years (95% CI), age- & sex-adjusted	*P*
BMI categories							
UW	62	29	46.8	75.75 (52.64–109.01)	0.008	21.11 (14.47–30.80)	0.025
NW	3349	921	27.5	38.35 (35.95.40.90)	Ref.	12.26 (10.67–14.08)	Ref.
OW	6569	1433	21.8	29.24 (27.77–30.80)	< 0.0001	10.39 (9.10–11.86)	< 0.0001
Ob I	3842	824	21.4	28.42 (26.55–30.43)	< 0.0001	11.70 (10.25–13.36)	0.339
Ob II	1312	276	21.0	28.01 (24.89–31.52)	< 0.0001	14.80 (12.65–17.30)	0.0101
Ob III	522	119	22.8	30.87 (25.79–36.94)	0.0016	20.67 (16.88–25.30)	< 0.0001
WC tertiles							
I	1526	265	17.4	22.14 (19.63–24.97)	Ref.	12.17 (9.40–15.76)	Ref.
II	1527	252	16.5	20.57 (18.18–23.28)	0.404	10.28 (7.93–13.33)	0.056
III	1525	352	23.1	29.92 (26.95–33.21)	< 0.0001	15.97 (12.43–20.51)	0.001
WHtR tertiles							
I	1528	240	15.7	19.75 (17.41–22.42)	Ref.	11.62 (8.96–15.07)	Ref.
II	1526	253	16.6	20.73 (18.33–23.45)	0.593	10.88 (8.40–14.08)	0.463
III	1524	376	24.7	32.33 (29.22–35.77)	< 0.0001	16.21 (12.61–20.83)	< 0.0001
ABSI tertiles							
I	1527	219	14.3	17.81 (15.60–20.34)	Ref.	12.07 (9.31–15.65)	Ref.
II	1527	258	16.9	21.21 (18.77–23.96)	0.057	11.75 (9.09–15.19)	0.918
III	1524	392	25.7	34.01 (30.80–37.55)	< 0.0001	14.93 (11.58–19.24)	0.013

BMI: body mass index; WC: waist circumference; WHtR: waist-to-height ratio; ABSI: A Body Shape Index; UW: underweight; NW: normal weight; OW: overweight; Ob-I: grade I obesity; Ob-II: grade II obesity; Ob-III: grade III obesity; CI: confidence interval

### Clinical features and mortality by surrogate measures of central adiposity

The clinical features of participants with WC measurements are shown in Additional file 6: Table S3. As compared with those without WC measurements, they were younger and had lower diabetes duration and HbA_1c_ levels than those without. As a consequence, they had also lower prevalence of complications (except DR) and death (19.0 vs 24.7%, *P* < 0.0001) and lower unadjusted death rate (24.15 [22.60–25.81] vs 34.11 [32.86–35.42] per 1,000 patient-years, *P* < 0.0001) and mortality risk (0.704 [0.652–0.760], *P* < 0.0001) than participants without WC measurements. However, both death rate (12.56 [11.02–14.32] vs 12.76 [11.30–14.41] per 1,000 patient-years, *P* = 0.697) and mortality risk (0.977 [0.904–1.057], *P* = 0.563) became similar after adjusting for age and sex.

The three measures of central adiposity were significantly correlated between each other and with BMI. Correlations were stronger between WC and WHtR than between either one and ABSI; compared with WC and WHtR, correlation of ABSI with BMI was weaker and inverse (Additional file 7: Table S4).

The clinical features of the RIACE participants stratified by WC, WHtR, and ABSI tertiles are shown in in Additional file 8: Table S5, respectively. Individuals in tertile III were slightly older, more frequently current smokers, with lower PA level (except for ABSI), LDL cholesterol, and eGFR and higher HbA_1c_, triglycerides, triglycerides:HDL ratio, systolic BP, albuminuria, and prevalence of hypertension, insulin, anti-hypertensive, anti-platelet, and anti-coagulant treatment, DKD, DR, CVD, and comorbidities, especially COPD, compared to those in tertile I of each measure. Moreover, BMI increased and HDL cholesterol decreased from tertile I to tertile III of WC and WHtR, whereas the opposite trend was observed for ABSI.

**Fig. 3:**
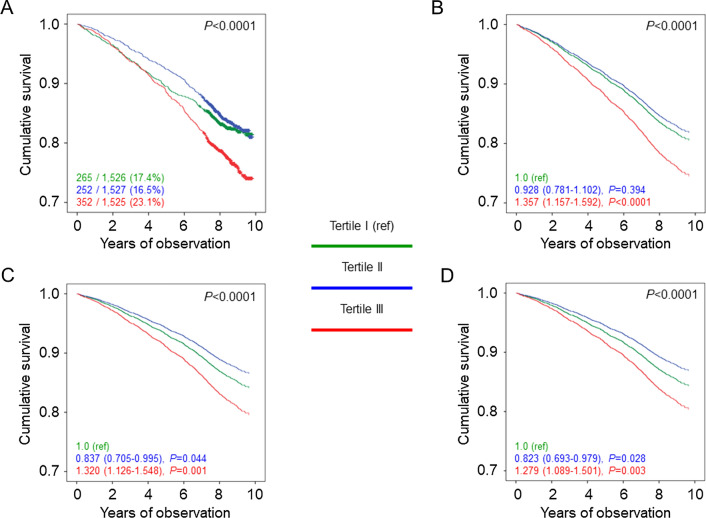
Survival analysis by WC tertiles. Kaplan–Meier analysis (**A**) and Cox proportional hazards regression, unadjusted (**B**) and adjusted for age and sex (**C**), and age, sex, smoking status, PA level, and comorbidities (D), according to WC tertiles. Numbers (percentages) of deaths and HRs (95% CI) for mortality are shown for each group. WC: waist circumference; PA: physical activity; HR: hazard ratio; CI: confidence interval

**Fig. 4:**
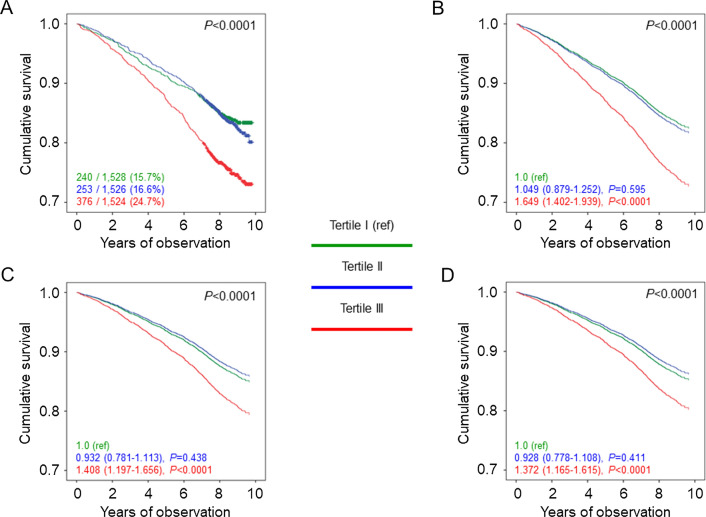
Survival analysis by WHtR tertiles. Kaplan Meier analysis (**A**) and Cox proportional hazards regression, unadjusted (**B**) and adjusted for age and sex (**C**), and age, sex, smoking status, PA level, and comorbidities (**D**), according to WHtR tertiles. Numbers (percentages) of deaths and HRs (95% CI) for mortality are shown for each group. WHtR: waist-to-height ratio; PA: physical activity; HR: hazard ratio; CI: confidence interval

**Fig. 5:**
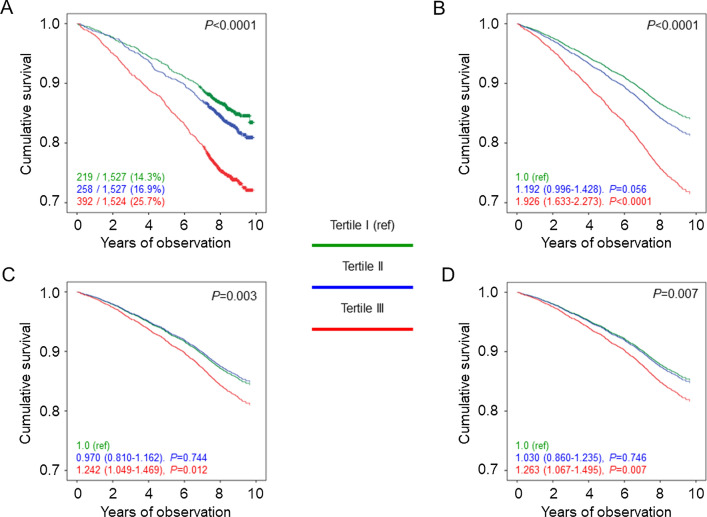
Survival analysis by ABSI tertiles. Kaplan–Meier analysis (**A**) and Cox proportional hazards regression, unadjusted (**B**) and adjusted for age and sex (**C**), and age, sex, smoking status, PA level, and comorbidities (**D**), according to ABSI tertiles. Numbers (percentages) of deaths and HRs (95% CI) for mortality are shown for each group. ABSI: A Body Shape Index; PA: physical activity; HR: hazard ratio; CI: confidence interval

Percent deaths (Additional file 8: Table S5), crude and age- and sex-adjusted mortality rates (Table 1), Kaplan–Meier estimates (Figs. 3A, 4A, and 5A), and unadjusted HRs (Figs. 3B, 4B, and 5B) were higher in tertile III versus tertile I of each measure. Significant differences remained when adjusting for age and sex (Figs. 3C, 4C, and 5C) and when further adjusting for smoking, PA level, and comorbidities (Figs. 3D, 4D, and 5D) and, except for WC, for CVD risk factors and complications (not shown). Moreover, HRs were significantly lower in tertile II versus tertile I of WC when sequentially adjusting for age, sex, smoking, PA level, and comorbidities (Fig. 3C, D) and even after further adjustment for CVD risk factors and complications (not shown), whereas no significant differences were detected between tertile II and tertile I of WHtR (Fig. 4C, D) and an opposite, though non-significant trend was observed for ABSI (Fig. 5C, D). Further adjustment for BMI did not affect the association of WC and WHtR with mortality (not shown). Results of the analyses conducted separately in men and women yielded the same results as in the whole cohort for WC and WHtR tertiles, whereas the linear relationship between ABSI tertiles and death was evident only in males (Additional file 5: Table S2). Moreover, the associations between WC or WHtR and mortality were unaffected by further adjustment for BMI in males, whereas they disappeared in females (not shown).

## Discussion

By comparing BMI and three surrogate indices of central adiposity as measure of obesity, this analysis provides important insights into the obesity paradox for mortality in patients with type 2 diabetes. On the one hand, the paradox relationship between BMI and death was limited to the overweight category, with the individuals with grade I obesity showing no survival advantage compared to those in the normal-weight range. On the other hand, an obesity paradox was also observed when adiposity was defined using WC, but not WHtR and especially ABSI, instead of BMI.

The nadir for mortality risk in the overweight range is consistent with previous studies in people with type 2 diabetes [16–19], though other surveys showed a nadir for mortality well above the obesity threshold [20–24]. Furthermore, a meta-analysis of 414,587 participants with type 2 diabetes reported that all-cause mortality was lowest in those with a BMI in the overweight and obesity range in women and men, respectively [15], and a pooled analysis of 5 longitudinal cohort studies showed a lower adjusted risk of death in overweight/obese than in normal-weight adults with incident diabetes [14]. However, a survival benefit for overweight individuals was also reported in a meta-analysis of 97 prospective studies of general populations of adults [33] and in both diabetic and non-diabetic individuals from the US National Health Interview Survey [16], pointing to the existence of a BMI paradox rather than an obesity/overweight paradox.

The unchanged relationship between BMI and mortality after controlling for age, smoking, and comorbidities argues against a confounding from collider bias or reverse causation. The same BMI-mortality curve as in the entire cohort was in fact obtained after sequentially excluding ever smokers, patients with comorbidities, and those who died within 2 years of follow-up and when analyzing separately participants below and above the median age. In addition, when further adjusting for smoking, PA level, and comorbidities, risk of death versus normal-weight individuals became significantly lower also in those in the mildly obese range. This is at variance with reports from the general population or people with type 2 diabetes showing no obesity paradox when considering factors potentially affecting this association. In fact, an inverse relationship between BMI and death was found in diabetic individuals aged ≥ 65 years and a direct one in those aged < 65 years [34]. Moreover, previous studies showed an obesity paradox among ever smokers, but not never smokers [35, 36], and an attenuated or no obesity paradox when restricting the analysis to never smokers without previous disease who survived at least 5 or 3 years, respectively [1, 16]. However, a study in patients with type 2 diabetes also reported that the association of BMI and central adiposity measures with mortality did not change when excluding individuals with cancer or surviving at least one year [28].

The U-shaped BMI-mortality curve observed in inactive or moderately inactive participants, but not in those accumulating moderate or high amounts of PA, suggests that the obesity (overweight) paradox might be explained, at least partly, by two unmeasured PA-related confounders that are rarely accounted for in studies assessing the relationship between obesity and mortality, i.e., physical fitness and body composition. In this view, underweight and even normal-weight individuals with a low PA level showed an increased risk of death compared to overweight patients because their lower body weight was also due to reduced muscle mass and was associated with poor cardiorespiratory fitness. Vice versa, overweight individuals with a high PA level showed a decreased risk of death compared to normal-weight patients because their higher body weight was also due to preserved muscle mass and was associated with good cardiorespiratory fitness. Our findings are consistent with previous studies investigating the relationship of cardiorespiratory fitness and lean (muscle) mass with death. The obesity paradox was in fact shown in patients with low but not in those with high cardiorespiratory fitness, regardless of using BMI, percent body fat or WC as a measure of adiposity [37]. Moreover, low fitness and obesity were independently and cumulatively associated with increased mortality [20] and fitness modified the effect of fatness to produce the obesity paradox [38], with individuals with good fitness and high BMI showing a lower risk for all-cause and CVD mortality than those with poor fitness and normal BMI [39]. Low lean mass, rather than low fat mass, was responsible for the increased mortality among individuals with low to normal BMI [40]. Furthermore, in older patients with heart failure, low BMI was a better indicator of reduction in lean mass than of reduction in fat mass [41], and, in patients with cancer, an obesity paradox emerged when using BMI, but was not confirmed by analyses based on body composition [42]. However, muscle mass (and its contribution to body weight/BMI and body composition) does not necessarily reflect muscle strength (i.e., muscular fitness), which is a better marker of muscle quality than mass and a major factor influencing mortality independently of cardiorespiratory fitness [37].

Although muscle mass and function and cardiorespiratory fitness may have contributed to the overweight paradox in the RIACE cohort, a potential role for moderate adiposity in providing a survival benefit cannot be ruled out. In patients with stable coronary heart disease, both lean and fat mass were in fact shown to predict death, with the highest mortality in those with low lean and fat mass and the lowest mortality in those with high lean and fat mass and one condition being protective independently of the other [43]. Moreover, subcutaneous adipose tissue may be protective in cancer due to better nutritional status, with moderate amounts enabling patients to survive longer weight losses that can occur with tumour progression and treatment [44].

The lower mortality risk in the intermediate versus the lowest WC tertile seems to suggest a protective effect of moderate adiposity even at the visceral level. This at variance with previous reports using WC and/or WHR. A systematic review of the literature and collaborative analysis with individual subject data did in fact show that central obesity, as defined on the basis of WC and WHR tertiles, was associated with higher mortality in individuals with either normal or elevated BMI, though the impact of each one alone was significant only for WHR [11]. Moreover, in the Melbourne Collaborative Cohort Study, WC and WHR were stronger predictors of mortality than BMI and fat mass [45], though other studies using measures of fat distribution failed to show a linear relationship between these measures and death [46]. Contrasting data were also found in previous studies in diabetic individuals, as WC and WHR were shown to be either associated [27] or not associated [47] with mortality. However, in our study, the difference between the lowest and intermediate tertile was not significant when using WHtR and tended to be inverted with ABSI. These data are consistent with previous reports showing that either WHtR [27] or ABSI [28] were superior to WC and WHR in predicting mortality. Moreover, the findings that the associations between WC or WHtR and mortality disappeared after adjusting for BMI in females and the relationship between ABSI and mortality was stronger in men than in women are also consistent with previous reports in people with type 2 diabetes [27, 28] and can be due to sex differences in body fat distribution [48].

Our findings have relevant implications for risk stratification and weight management strategies in individuals with type 2 diabetes. First, surrogate measures of central adiposity that normalize WC to height (and BMI) are superior to WC in integrating BMI for outcome prediction, as they separate the influence of the component of body shape measured by WC and reflecting visceral fat distribution from that of body size. Therefore, in routine clinical practice, the calculation of WHtR and especially ABSI from WC is highly recommended for prognostic purposes in people with type 2 diabetes in order to account for the detrimental impact of mild increases in (central) body fat. Second, though a moderate level of subcutaneous fat might confer a survival advantage to individuals getting older or developing potentially fat illnesses, this does not imply that weight loss programs are not indicated in overweight and mildly obese patients with type 2 diabetes, especially if they have excess central fat accumulation. Instead, our data support the need for combined diet and exercise intervention in order to minimize loss of muscle (and bone) mass associated with diet only [49], which may have a detrimental impact on mortality. However, further studies with concurrent assessment of body composition, fat distribution, and physical fitness are needed to dissect the relative contribution of these variables to survival in elderly individuals and patients suffering from chronic disorders and inform effective strategies for disease management.

Strength of our study include the comparison of BMI and three surrogate measures of central adiposity (including both WHtR and ABSI), the large sample size, the assessment of a wide range of clinical parameters, the completeness of baseline and follow-up data. However, this study has several limitations. First, WC and WC-derived measures (WHtR and ABSI) were available only from 4618 individuals, though adjusted death rate and mortality risk were similar to those of participants without these measurements. Second, lack of information about the weight history of the RIACE participants both before and after enrolment may have influenced the results, though the potential impact of reverse causation from unintentional weight loss due to undiagnosed or diagnosed illnesses was reduced by excluding patients with comorbidities and deceased during the first two years of follow-up. Third, relevant confounders such as body composition and physical fitness were not assessed, and PA level, which might be considered a surrogate measure of these parameters, was self-reported. Fourth, the study findings may not be applicable to the general ambulatory diabetes population, as only part of the individuals with type 2 diabetes attend Diabetes Clinics in Italy; however, the RIACE cohort is representative of patients followed by diabetes specialists in these clinics [50]. Finally, the observational design makes causal interpretation impossible.

## Conclusions

In patients with type 2 diabetes from the RIACE cohort, the lowest mortality risk was in the overweight BMI range. This overweight paradox could not be explained by confounders such as age, smoking, and comorbidities, but it could be attributed, at least partly, to confounding from PA level, likely through its impact on lean (muscle) mass and cardiorespiratory fitness, in addition to a possible survival advantage from a moderate excess of peripheral fat.

Intermediate values of WC were associated with a lower risk of death compared to low values, but a protective effect of moderate central fat accumulation was not confirmed by using WHtR and especially ABSI (at least in men) as surrogate measures of adiposity, which better reflected mortality risk associated with central adiposity and, therefore, should be routinely assessed in people with type 2 diabetes for prognostic purposes.

## Supplementary Information

**Additional file 1.** The RIACE Study Group.

**Additional file 2: Table S1.** Baseline clinical features of study participants by BMI categories.

**Additional file 3: Figure S1.** Survival analysis by age categories.

**Additional file 4: Figure S2.** Survival analysis after sequential patients’ exclusion.

**Additional file 5: Table S2.** Survival analysis by BMI categories according to sex.

**Additional file 6: Table S3.** Baseline clinical features of study participants as a whole and by availability of WC measurements.

**Additional file 7: Table S4.** Univariate correlations of surrogate measures of central adiposity between each other and with BMI by Pearson correlation coefficient.

**Additional file 8: Table S5.** Baseline clinical features of study participants by WC, WHtR, and ABSI tertiles.

## Data Availability

The datasets used and/or analysed during the current study are available from the corresponding author on reasonable request.

## Abbreviations

BMI: Body mass index
BP: Blood pressure
CI: Confidence interval
CVD: Cardiovascular disease
DKD: Diabetic kidney disease
DR: Diabetic retinopathy
eGFR: Estimated glomerular filtration rate
HbA_1c_: Haemoglobin A_1c_
PA: Physical activity
RIACE: Renal Insufficiency And Cardiovascular Events
WHtR: Waist-to-height ratio
WC: Waist circumference
WHR: Waist-to-hip ratio
