# Supplementary material for: Body mass index versus surrogate measures of central adiposity as independent predictors of mortality in type 2 diabetes
Source: Cardiovasc Diabetol. 2022 Dec 2;21:266. doi: 10.1186/s12933-022-01706-2 (PMC9716975; doi:10.1186/s12933-022-01706-2)
Supplement: Supplementary file 2 — Additional file 2: Table S1. Baseline clinical features of study participants by BMI categories. [file 12933_2022_1706_MOESM2_ESM.doc]

**Additional file 2: Table S1.** Baseline clinical features of study participants by BMI categories.

| **Variables** | **BMI categories** | | | | | | ***P*** |
| --- | --- | --- | --- | --- | --- | --- | --- |
|  | **UW** | **NW** | **OW** | **Ob-I** | **Ob-II** | **Ob-III** |  |
| **N (%)** | 62 (0.4) | 3,349 (21.4) | 6,569 (42.0) | 3,842 (24.5) | 1,312 (8.4) | 522 (3.3) |  |
| **BMI, kg·m^-2^** | 17.7±0.7 | 23.1±1.5 | 27.5±1.4 | 32.1±1.4 | 37.0±1.4 | 43.9±4.0 |  |
| **(range)** | (16.1-18.5) | (18.5-25.0) | (25.0-30.0) | (30.0-35.0) | (35.0-40.0) | (40.0-67.9) |  |
| **Deaths, n (%)** | 29 (46.8) | 921 (27.5) | 1,433 (21.8) | 824 (21.4) | 276 (21.0) | 119 (22.8) | <0.0001 |
| **Age, years** | 69.9±13.8 | 68.1±11.0 | 67.1±10.1 | 65.9±9.9 | 63.9±9.9 | 61.5±10.0 | <0.0001 |
| **Sex, n (%)** |  |  |  |  |  |  | <0.0001 |
| **Females** | 44 (71.0) | 1,366 (40.8) | 2421 (36.9) | 1,780 (46.3) | 774 (59.0) | 369 (70.7) |  |
| **Males** | 18 (29.0) | 1,983 (58.2) | 4,148 (63.1) | 2,062 (53.7) | 538 (41.0) | 153 (29.3) |  |
| **Smoking, n (%)** |  |  |  |  |  |  | <0.0001 |
| **Never** | 42 (67.7) | 1,975 (59.0) | 3,582 (54.5) | 2,114 (55.0) | 799 (60.9) | 337 (64.6) |  |
| **Former** | 8 (12.9) | 823 (24.6) | 1,953 (29.7) | 1,165 (30.3) | 338 (25.8) | 120 (23.0) |  |
| **Current** | 12 (19.4) | 551 (16.5) | 1,034 (15.7) | 563 (14.7) | 175 (13.3) | 65 (12.5) |  |
| **Physical activity, n (%)** |  |  |  |  |  |  | <0.0001 |
| **inactive or moderately inactive** | 37 (59.7) | 1,923 (57.4) | 4,023 (61.2) | 2,608 (67.9) | 947 (72.2) | 406 (77.8) |  |
| **moderately active** | 25 (40.3) | 1,353 (40.4) | 2,446 (37.2) | 1,188 (30.9) | 350 (26.7) | 114 (21.8) |  |
| **highly active** | 0 (0.0) | 73 (2.2) | 100 (1.5) | 46 (1.2) | 15 (1.1) | 2 (0.4) |  |
| **Diabetes duration, years** | 15.4±11.1 | 14.7±10.9 | 13.4±10.1 | 12.4±9.8 | 11.5±9.3 | 11.1±9.4 | <0.0001 |
| **HbA_1c_, %** | 7.33±1.49 | 7.45±1.49 | 7.45±1.42 | 7.64±1.54 | 7.81±1.64 | 7.96±1.70 | <0.0001 |
| **Triglycerides, mmol·l^-1^** | 1.11±0.63 | 1.28±0.84 | 1.56±0.99 | 1.75±1.08 | 1.79±1.00 | 1.77±0.83 | <0.0001 |
| **Total cholesterol, mmol·l^-1^** | 4.70±0.96 | 4.75±0.99 | 4.77±0.99 | 4.82±1.00 | 4.86±0.97 | 4.78±0.95 | 0.001 |
| **HDL cholesterol, mmol·l^-1^** | 1.61±0.46 | 1.38±0.38 | 1.28±0.34 | 1.25±0.34 | 1.25±0.33 | 1.24±0.34 | <0.0001 |
| **Triglycerides:HDL ratio** | 1.85±1.68 | 2.46±2.57 | 134.6±36.7 | 137.9±37.3 | 139.5±36.1 | 136.9±34.7 | <0.0001 |
| **Non-HDL cholesterol, mmol·l^-1^** | 3.09±0.83 | 3.37±0.94 | 3.49±0.95 | 3.57±0.97 | 3.61±0.93 | 3.55±0.90 | <0.0001 |
| **LDL cholesterol, mmol·l^-1^** | 2.58±0.76 | 2.79±0.84 | 2.79±0.85 | 2.79±0.86 | 2.81±0.83 | 2.74±0.81 | 0.306 |
| **Dyslipidaemia, n (%)** | 45 (72.6) | 2,612 (78.0) | 5,457 (83.1) | 3,247 (84.5) | 1,076 (82.0) | 419 (80.3) | <0.0001 |
| **Systolic BP, mmHg** | 132.3±21.5 | 135.7±18.5 | 138.0±17.7 | 139.6±17.7 | 139.7±18.2 | 139.9±18.6 | <0.0001 |
| **Diastolic BP, mmHg** | 76.2±9.9 | 76.9±9.4 | 78.5±9.1 | 80.0±9.4 | 80.6±10.2 | 80.8±10.2 | <0.0001 |
| **Pulse pressure, mmHg** | 56.0±19.2 | 58.8±16.0 | 59.4±15.7 | 59.7±15.5 | 59.1±15.8 | 59.1±14.9 | 0.095 |
| **Hypertension, n (%)** | 45 (72.6) | 2,526 (75.4) | 5,443 (82.9) | 3,424 (89.1) | 1,184 (90.2) | 474 (90.8) | <0.0001 |
| **Anti-hyperglycaemic treatment, n (%)** |  |  |  |  |  |  | <0.0001 |
| **Lifestyle** | 8 (12.9) | 506 (15.1) | 959 (14.6) | 454 (11.8) | 133 (10.1) | 53 (10.2) |  |
| **Non-insulin** | 33 (53.2) | 2,037 (60.8) | 4,064 (61.9) | 2,362 (61.5) | 811 (61.8) | 312 (59.8) |  |
| **Insulin** | 21 (33.9) | 806 (24.1) | 1,546 (23.5) | 1,026 (26.7) | 368 (28.0) | 157 (30.1) |  |
| **Lipid-lowering treatment, n (%)** | 18 (29.0) | 1,373 (41.0) | 3,151 (48.0) | 1,895 (49.3) | 581 (44.3) | 220 (42.1) | <0.0001 |
| **Anti-hypertensive treatment, n (%)** | 33 (53.2) | 2,005 (59.9) | 4,538 (69.1) | 2,999 (78.1) | 1,068 (81.4) | 429 (82.2) | <0.0001 |
| **Anti-platelet treatment, n (%)** | 25 (40.3) | 1,243 (37.1) | 2,632 (40.1) | 1,644 (42.8) | 502 (38.3) | 202 (38.7) | <0.0001 |
| **Anti-coagulant treatment, n (%)** | 1 (1.6) | 128 (3.8) | 275 (4.2) | 170 (4.4) | 63 (4.8) | 32 (6.1) | 0.137 |
| **Albuminuria, mg·day^-1^** | 111.5±372.8 | 53.7±227.5 | 67.6±329.3 | 90.1±334.0 | 78.8±249.6 | 99.6±564.1 | <0.0001 |
| **Serum creatinine, μmol·l^-1^** | 68.1±22.3 | 79.2±30.5 | 82.6±37.1 | 81.7±33.2 | 78.7±31.5 | 77.6±39.8 | <0.0001 |
| **eGFR, ml·min^-1^·1.73m^-2^** | 83.2±21.4 | 80.9±20.4 | 79.8±20.4 | 79.6±21.4 | 81.2±21.9 | 83.1±24.4 | <0.0001 |
| **DKD phenotype, n (%)** |  |  |  |  |  |  | <0.0001 |
| **No DKD** | 37 (59.7) | 2,288 (68.3) | 4,249 (64.7) | 2,299 (59.8) | 797 (60.7) | 314 (60.2) |  |
| **Albuminuric DKD with preserved eGFR** | 16 (25.8) | 517 (15.4) | 1,192 (18.1) | 837 (21.8) | 289 (22.0) | 117 (22.4) |  |
| **Nonalbuminuric DKD** | 6 (9.7) | 313 (9.3) | 595 (9.1) | 389 (10.1) | 124 (9.5) | 49 (9.4) |  |
| **Albuminuric DKD with reduced eGFR** | 3 (4.8) | 233 (7.0) | 533 (8.1) | 317 (8.3) | 102 (7.8) | 42 (8.0) |  |
| **DR, n (%)** |  |  |  |  |  |  | 0.005 |
| **No DR** | 47 (75.8) | 2,671 (79.8) | 5,138 (78.2) | 2,946 (76.7) | 982 (74.8) | 405 (77.6) |  |
| **Non-advanced DR** | 10 (16.1) | 407 (12.2) | 801 (12.2) | 494 (12.9) | 176 (13.4) | 59 (11.3) |  |
| **Advanced DR** | 5 (8.1) | 271 (8.1) | 630 (9.6) | 402 (10.5) | 154 (11.7) | 58 (11.1) |  |
| **CVD, n (%)** |  |  |  |  |  |  |  |
| **Any** | 10 (16.1) | 748 (22.3) | 1,559 (23.7) | 939 (24.4) | 267 (20.4) | 97 (18.6) | 0.001 |
| **Myocardial infarction** | 6 (9.7) | 361 (10.8) | 751 (11.4) | 464 (12.1) | 130 (9.9) | 30 (5.7) | 0.001 |
| **Coronary revascularization** | 4 (6.5) | 314 (9.4) | 686 (10.4) | 412 (10.7) | 125 (9.5) | 38 (7.3) | 0.065 |
| **Any coronary event** | 7 (11.3) | 505 (14.9) | 1,029 (15.7) | 628 (16.3) | 178 (13.6) | 56 (10.7) | 0.005 |
| **Stroke** | 1 (1.6) | 103 (3.1) | 232 (3.5) | 139 (3.6) | 28 (2.1) | 10 (1.9) | 0.032 |
| **Carotid revascularization** | 2 (3.2) | 189 (5.6) | 352 (5.4) | 218 (5.7) | 69 (5.3) | 26 (5.0) | 0.899 |
| **Any carotid event** | 3 (4.8) | 271 (8.1) | 554 (8.4) | 336 (8.7) | 93 (7.1) | 35 (6.7) | 0.253 |
| **Ulcer/gangrene/amputation** | 3 (4.8) | 114 (3.3) | 232 (3.5) | 137 (3.6) | 50 (3.8) | 23 (4.4) | 0.816 |
| **Lower limb revascularization** | 1 (1.6) | 100 (3.0) | 214 (3.3) | 107 (2.8) | 22 (1.7) | 7 (1.3) | 0.010 |
| **Any peripheral event** | 3 (4.8) | 180 (5.4) | 390 (5.9) | 215 (5.6) | 66 (5.0) | 29 (5.6) | 0.770 |
| **Comorbidities n (%)** |  |  |  |  |  |  |  |
| **Any** | 18 (29.0) | 606 (18.1) | 1,110 (16.9) | 699 (18.2) | 259 (19.7) | 95 (18.2) | 0.023 |
| **COPD** | 2 (3.2) | 129 (3.9) | 236 (3.6) | 185 (4.8) | 80 (6.1) | 42 (8.0) | <0.0001 |
| **Chronic liver disease** | 8 (12.9) | 316 (9.4) | 555 (8.4) | 318 (8.3) | 121 (9.2) | 43 (8.2) | 0.343 |
| **Cancer** | 11 (17.7) | 231 (6.9) | 411 (6.3) | 270 (7.0) | 83 (6.3) | 25 (4.8) | 0.003 |

BMI = body mass index; UW = underweight; NW = normal weight; OW = overweight; Ob-I = grade I obesity; Ob-II = grade II obesity; Ob-III = grade III obesity; HbA_1c_ = haemoglobin A_1c_; BP = blood pressure; eGFR = estimated glomerular filtration rate; DKD = diabetic kidney disease; DR = diabetic retinopathy; = CVD = cardiovascular disease; COPD = chronic obstructive pulmonary disease.
