# Supplementary material for: Body mass index versus surrogate measures of central adiposity as independent predictors of mortality in type 2 diabetes
Source: Cardiovasc Diabetol. 2022 Dec 2;21:266. doi: 10.1186/s12933-022-01706-2 (PMC9716975; doi:10.1186/s12933-022-01706-2)
Supplement: Supplementary file 3 — Additional file 3: Figure S1. Survival analysis by age categories. [file 12933_2022_1706_MOESM3_ESM.doc]

**
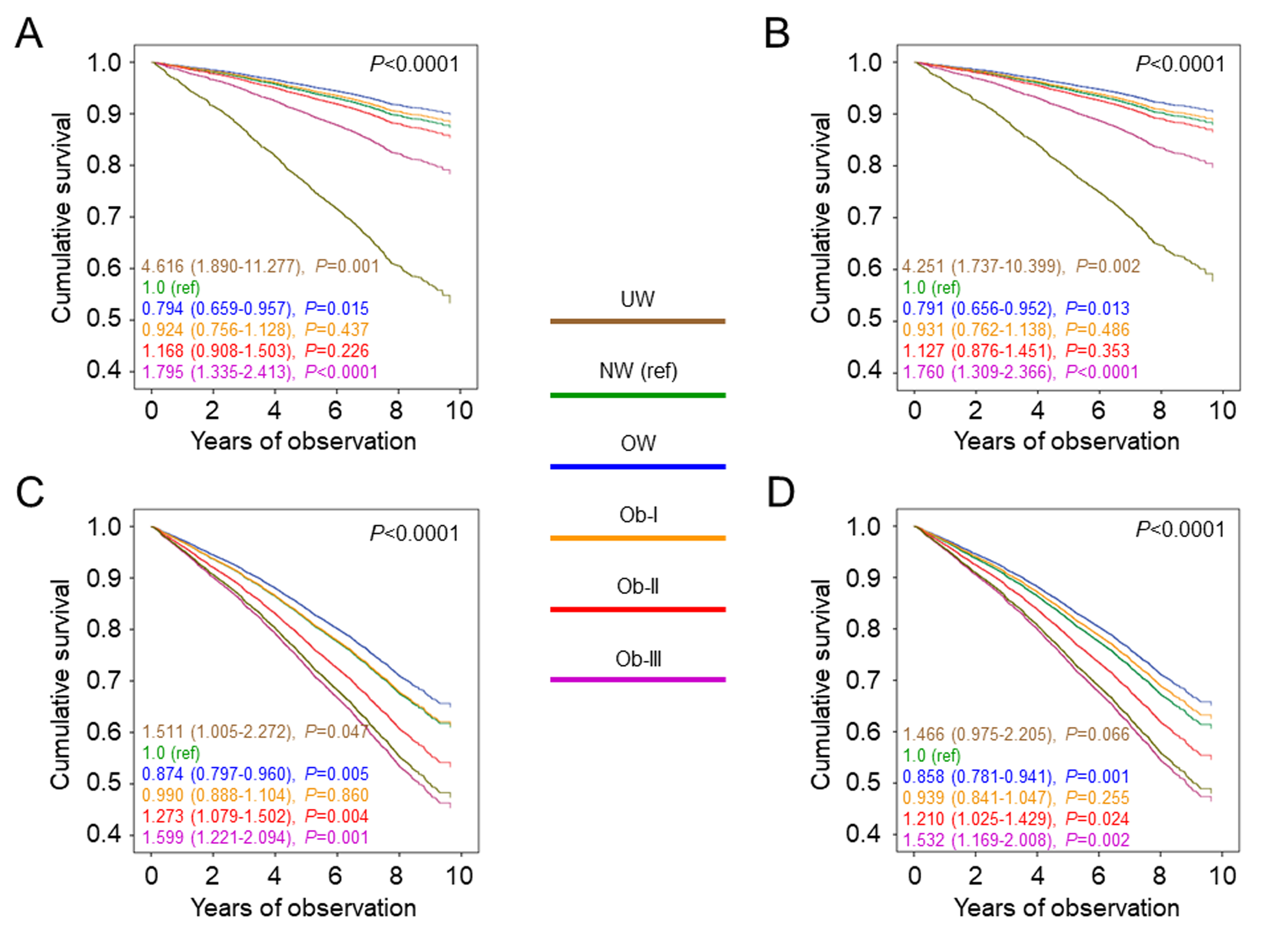
**

**Additional file 3: Figure S1.** Survival analysis by age categories. Cox proportional hazards regression and adjusted for age and sex (A, C) and age, sex, smoking status, PA level, and comorbidities (B, D), according to BMI categories, in patients below (A, B) and above (C, D) median age. HRs (95% CI) for mortality are shown for each group. PA = physical activity; BMI = body mass index; HR = hazard ratio; CI = confidence interval; UW = underweight; NW = normal-weight; OW = overweight; Ob-I = grade I obesity; Ob-II = grade II obesity; Ob-III = grade III obesity.
