# Supplementary material for: Body mass index versus surrogate measures of central adiposity as independent predictors of mortality in type 2 diabetes
Source: Cardiovasc Diabetol. 2022 Dec 2;21:266. doi: 10.1186/s12933-022-01706-2 (PMC9716975; doi:10.1186/s12933-022-01706-2)
Supplement: Supplementary file 4 — Additional file 4: Figure S2. Survival analysis after sequential patients’ exclusion. [file 12933_2022_1706_MOESM4_ESM.doc]

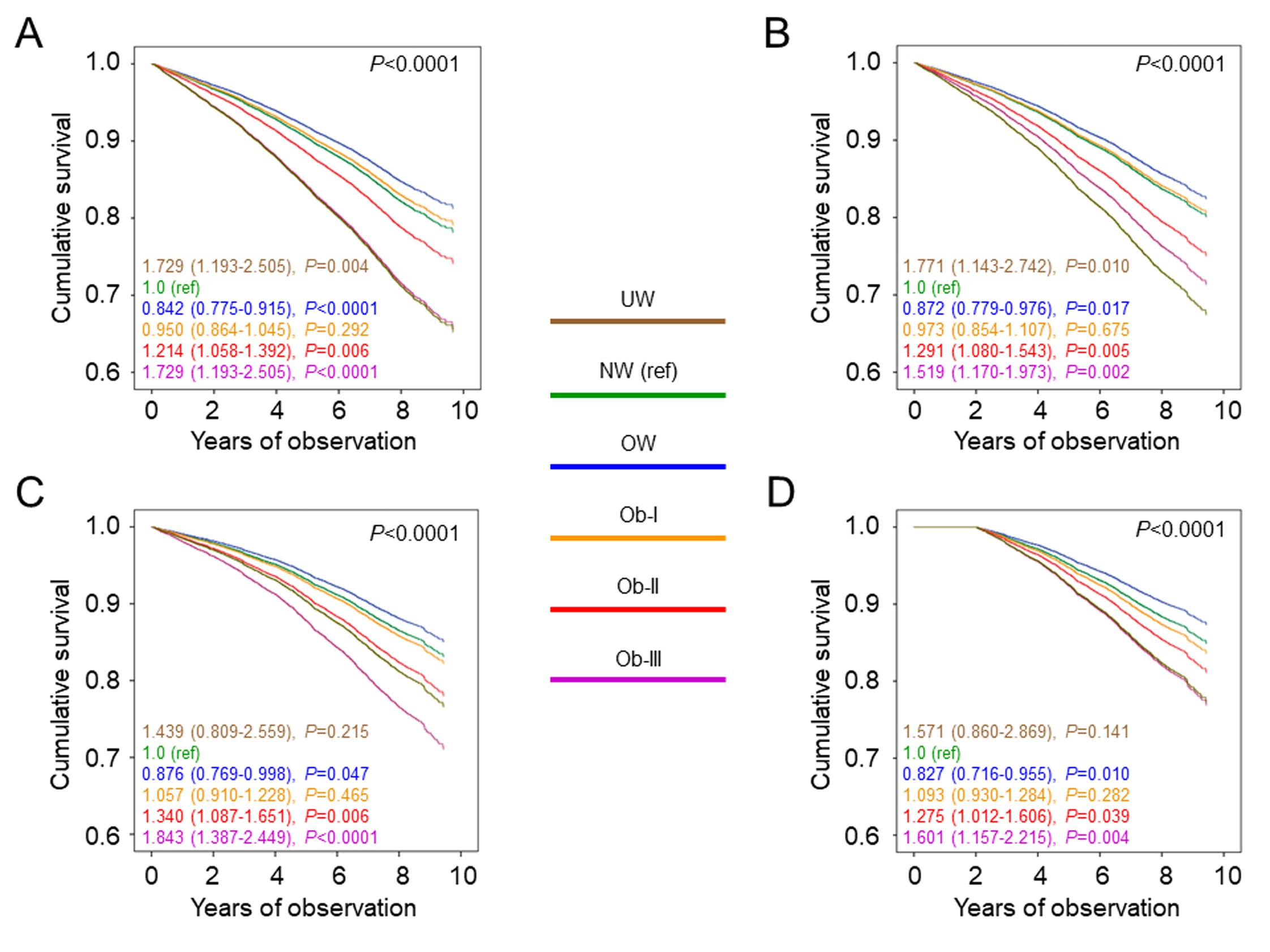


**Additional file 4: Figure S2.** Survival analysis after sequential patients’ exclusion. Cox proportional hazards regression, adjusted for age and sex, according to BMI categories, in the whole cohort (A) and after sequentially excluding former and current smokers (B), patients with severe comorbidities (C), and those who died within two years since enrolment (D). HRs (95% CI) for mortality are shown for each group. BMI = body mass index; HR = hazard ratio; CI = confidence interval; UW = underweight; NW = normal-weight; OW = overweight; Ob-I = grade I obesity; Ob-II = grade II obesity; Ob-III = grade III obesity.
