# Supplementary material for: Body mass index versus surrogate measures of central adiposity as independent predictors of mortality in type 2 diabetes
Source: Cardiovasc Diabetol. 2022 Dec 2;21:266. doi: 10.1186/s12933-022-01706-2 (PMC9716975; doi:10.1186/s12933-022-01706-2)
Supplement: Supplementary file 5 — Additional file 5: Table S2. Survival analysis by BMI categories according to sex. [file 12933_2022_1706_MOESM5_ESM.doc]

**Additional file 5: Table S2.** Survival analysis by BMI categories according to sex. Cox proportional hazards regression, unadjusted and adjusted for age and sex (Model 1), and age, smoking status, PA level, and comorbidities (Model 2) by BMI categories and WC, WHtR, and ABSI tertiles, in all participants and in separately females and males.

| **BMI categories** | **Unadjusted** | | | **Model 1** | | | **Model 2** | | |
| --- | --- | --- | --- | --- | --- | --- | --- | --- | --- |
|  | **HR** | **95% CI** | ***P*** | **HR** | **95% CI** | ***P*** | **HR** | **95% CI** | ***P*** |
| **All** |  |  |  |  |  |  |  |  |  |
| **NW** | 1.000 | - | <0.0001 | 1.000 | - | <0.0001 | 1.000 | - | <0.0001 |
| **UW** | 2.006 | 1.386-2.904 | <0.0001 | 1.729 | 1.193-2.505 | 0.004 | 1.676 | 1.156-2.428 | 0.006 |
| **OW** | 0.760 | 0.700-0.826 | <0.0001 | 0.842 | 0.775-0.915 | <0.0001 | 0.828 | 0.762-0.900 | <0.0001 |
| **Ob-I** | 0.738 | 0.672-0.811 | <0.0001 | 0.950 | 0.864-1.045 | 0.292 | 0.907 | 0.824-0.998 | 0.045 |
| **Ob-II** | 0.728 | 0.636-0.833 | <0.0001 | 1.214 | 1.058-1.392 | 0.006 | 1.150 | 1.002-1.319 | 0.047 |
| **Ob-II** | 0.803 | 0.664-0.972 | 0.025 | 1.703 | 1.402-2.068 | <0.0001 | 1.613 | 1.327-1.961 | <0.0001 |
| **Females** |  |  |  |  |  |  |  |  |  |
| **NW** | 1.000 | - | <0.0001 | 1.000 | - | <0.0001 | 1.000 | - | <0.0001 |
| **UW** | 1.942 | 1.224-3.081 | 0.005 | 1.330 | 0.838-2.112 | 0.226 | 1.271 | 0.800-2.017 | 0.310 |
| **OW** | 0.675 | 0.587-0.775 | <0.0001 | 0.742 | 0.645-0.853 | <0.0001 | 0.737 | 0.641-0.847 | <0.0001 |
| **Ob-I** | 0.726 | 0.627-0.841 | <0.0001 | 0.902 | 0.778-1.046 | 0.174 | 0.875 | 0.754-1.015 | 0.078 |
| **Ob-II** | 0.698 | 0.576-0.844 | <0.0001 | 1.145 | 0.942-1.391 | 0.173 | 1.093 | 0.899-1.330 | 0.371 |
| **Ob-II** | 0.799 | 0.627-1.018 | 0.070 | 1.658 | 1.293-2.125 | <0.0001 | 1.592 | 1.240-2.043 | <0.0001 |
| **Males** |  |  |  |  |  |  |  |  |  |
| **NW** | 1.000 | - | <0.0001 | 1.000 | - | <0.0001 | 1.000 | - | <0.0001 |
| **UW** | 2.389 | 1.279-4.465 | 0.006 | 2.865 | 1.533-5.355 | 0.001 | 2.953 | 1.579-5.525 | 0.001 |
| **OW** | 0.803 | 0.724-0.890 | <0.0001 | 0.902 | 0.813-1.000 | 0.051 | 0.882 | 0.794-0.979 | 0.018 |
| **Ob-I** | 0.755 | 0.667-0.853 | <0.0001 | 0.977 | 0.863-1.106 | 0.714 | 0.923 | 0.814-1.045 | 0.206 |
| **Ob-II** | 0.800 | 0.659-0.970 | 0.023 | 1.262 | 1.038-1.533 | 0.019 | 1.194 | 0.982-1.453 | 0.075 |
| **Ob-II** | 0.890 | 0.643-1.231 | 0.480 | 1.694 | 1.223-2.347 | 0.002 | 1.608 | 1.160-2.231 | 0.004 |
| **WC tertiles** | **Unadjusted** | | | **Model 1** | | | **Model 2** | | |
|  | **HR** | **95% CI** | ***P*** | **HR** | **95% CI** | ***P*** | **HR** | **95% CI** | ***P*** |
| **All** |  |  |  |  |  |  |  |  |  |
| **I** | 1.000 | - | <0.0001 | 1.000 | - | <0.0001 | 1.000 | - | <0.0001 |
| **II** | 0.928 | 0.781.1.102 | 0.394 | 0.837 | 0.705-0.995 | 0.044 | 0.823 | 0.693-0.979 | 0.028 |
| **II** | 1.357 | 1.157-1.592 | <0.0001 | 1.320 | 1.126-1.548 | 0.001 | 1.279 | 1.089-1.501 | 0.003 |
| **Females** |  |  |  |  |  |  |  |  |  |
| **I** | 1.000 | - | <0.0001 | 1.000 | - | <0.0001 | 1.000 | - | <0.0001 |
| **II** | 0.940 | 0.716-1.236 | 0.659 | 0.835 | 0.636-1.098 | 0.197 | 0.840 | 0.639-1.104 | 0.210 |
| **II** | 1.471 | 1.147-1.888 | 0.002 | 1.401 | 1.091-1.797 | 0.008 | 1.394 | 1.086-1.789 | 0.009 |
| **Males** |  |  |  |  |  |  |  |  |  |
| **I** | 1.000 | - | 0.004 | 1.000 | - | 0.001 | 1.000 | - | 0.001 |
| **II** | 0.919 | 0.735-1.147 | 0.455 | 0.839 | 0.672-1.048 | 0.122 | 0.832 | 0.666-1.040 | 0.106 |
| **II** | 1.282 | 1.042-1.577 | 0.019 | 1.266 | 1.029-1.558 | 0.026 | 1.237 | 1.005-1.522 | 0.045 |
| **WHtR tertiles** | **Unadjusted** | | | **Model 1** | | | **Model 2** | | |
|  | **HR** | **95% CI** | ***P*** | **HR** | **95% CI** | ***P*** | **HR** | **95% CI** | ***P*** |
| **All** |  |  |  |  |  |  |  |  |  |
| **I** | 1.000 | - | <0.0001 | 1.000 | - | <0.0001 | 1.000 | - | <0.0001 |
| **II** | 1.049 | 0.879-1.252 | 0.595 | 0.932 | 0.781-1.113 | 0.438 | 0.928 | 0.778-1.108 | 0.411 |
| **II** | 1.649 | 1.402-1.939 | <0.0001 | 1.408-1.197 | 1.656 | <0.0001 | 1.372 | 1.165-1.615 | <0.0001 |
| **Females** |  |  |  |  |  |  |  |  |  |
| **I** | 1.000 | - | <0.0001 | 1.000 | - | <0.0001 | 1.000 | - | <0.0001 |
| **II** | 1.157 | 0.874-1.532 | 0.309 | 0.989 | 0.747-1.310 | 0.941 | 1.011 | 0.763-1.339 | 0.939 |
| **II** | 1.875 | 1.450-2.426 | <0.0001 | 1.546 | 1.194-2.001 | 0.001 | 1.568 | 1.211-2.032 | 0.001 |
| **Males** |  |  |  |  |  |  |  |  |  |
| **I** | 1.000 | - | <0.0001 | 1.000 | - | 0.001 | 1.000 | - | 0.002 |
| **II** | 0.983 | 0.783-1.234 | 0.882 | 0.897 | 0.714-1.126 | 0.347 | 0.891 | 0.710-1.118 | 0.319 |
| **II** | 1.510 | 1.226-1.861 | <0.0001 | 1.320 | 1.071-1.627 | 0.009 | 1.278 | 1.036-1.575 | 0.022 |
| **ABSI tertiles** | **Unadjusted** | | | **Model 1** | | | **Model 2** | | |
|  | **HR** | **95% CI** | ***P*** | **HR** | **95% CI** | ***P*** | **HR** | **95% CI** | ***P*** |
| **All** |  |  |  |  |  |  |  |  |  |
| **I** | 1.000 | - | <0.0001 | 1.000 | - | 0.003 | 1.000 | - | 0.007 |
| **II** | 1.192 | 0.996-1.428 | 0.056 | 0.970 | 0.810-1.162 | 0.744 | 1.030 | 0.860-1.235 | 0.746 |
| **II** | 1.926 | 1.633-2.273 | <0.0001 | 1.242 | 1.049-1.469 | 0.012 | 1.263 | 1.067-1.495 | 0.007 |
| **Females** |  |  |  |  |  |  |  |  |  |
| **I** | 1.000 | - | 0.002 | 1.000 | - | 0.231 | 1.000 | - | 0.209 |
| **II** | 0.990 | 0.755-1.297 | 0.941 | 0.793 | 0.605-1.040 | 0.094 | 0.828 | 0.630-1.087 | 0.173 |
| **II** | 1.448 | 1.126-1.860 | 0.004 | 0.923 | 0.716-1.190 | 0.535 | 1.027 | 0.792-1.332 | 0.841 |
| **Males** |  |  |  |  |  |  |  |  |  |
| **I** | 1.000 | - | <0.0001 | 1.000 | - | <0.0001 | 1.000 | - | 0.007 |
| **II** | 1.382 | 1.084-1.761 | 0.009 | 1.137 | 0.892-1.451 | 0.299 | 1.195 | 0.936-1.524 | 0.152 |
| **II** | 2.383 | 1.908-2.975 | <0.0001 | 1.557 | 1.241-1.954 | <0.0001 | 1.552 | 1.237-1.947 | <0.0001 |

PA = physical activity; HR = hazard ratio; CI = confidence interval; BMI = body mass index; NW = normal weight; UW = underweight; OW = overweight; Ob-I = grade I obesity; Ob-II = grade II obesity; Ob-III = grade III obesity; WC = waist circumference; WHtR = waist-to-height ratio; ABSI = A Body Shape Index.
