# Supplementary material for: Body mass index versus surrogate measures of central adiposity as independent predictors of mortality in type 2 diabetes
Source: Cardiovasc Diabetol. 2022 Dec 2;21:266. doi: 10.1186/s12933-022-01706-2 (PMC9716975; doi:10.1186/s12933-022-01706-2)
Supplement: Supplementary file 6 — Additional file 6: Table S3. Baseline clinical features of study participants as a whole and by availability of WC measurements. [file 12933_2022_1706_MOESM6_ESM.doc]

**Additional file 6: Table S3.** Baseline clinical features of study participants as a whole and by availability of WC measurements.

| **Variables** | **All cohort** | **With WC** | **Without WC** |
| --- | --- | --- | --- |
| **N** | 15,656 | 11,078 | 4,758 |
| **Deaths, n (%)** | 3,602 (23.0) | 2,733 (24.7) | 869 (19.0) |
| **Age, years** | 66.6±10.3 | 67.6±10.4 | 64.1±9.8 |
| **Sex, n (%)** |  |  |  |
| **Females** | 6,754 (43.1) | 4,802 (43.3) | 1,952 (42.6) |
| **Males** | 8,902 (56.9) | 6,276 (56.7) | 2,626 (57.4) |
| **Smoking, n (%)** |  |  |  |
| **Never** | 8,849 (56.5) | 6,398 (57.8) | 2,451 (53.5) |
| **Former** | 4,407 (28.1) | 3,098 (28.0) | 1,309 (28.6) |
| **Current** | 2,400 (15.3) | 1,582 (14.3) | 818 (17.9) |
| **Physical activity, n (%)** |  |  |  |
| **Inactive or moderately inactive** | 9,944 (63.5) | 7,062 (63.7) | 2,882 (63.0) |
| **Moderately active** | 5,476 (35.0) | 3,838 (34.6) | 1,638 (35.8) |
| **Highly active** | 236 (1.5) | 178 (1.6) | 58 (1.3) |
| **Diabetes duration, years** | 13.2±10.2 | 14.1±10.4 | 10.9±9.1 |
| **HbA_1c_, %** | 7.55±1.50 | 7.61±1.46 | 7.40±1.59 |
| **BMI, kg·m^-2^** | 29.0±5.1 | 28.7±5.1 | 29.6±5.3 |
| **Triglycerides, mmol·l^-1^** | 1.57±1.00 | 1.55±0.95 | 1.63±1.11 |
| **Total cholesterol, mmol·l^-1^** | 4.78±0.99 | 4.80±0.99 | 4.74±0.99 |
| **HDL cholesterol, mmol·l^-1^** | 1.29±0.35 | 1.29±0.35 | 1.29±0.36 |
| **Triglycerides:HDL ratio** | 3.18±2.76 | 3.14±2.7 | 3.28±2.8 |
| **Non-HDL cholesterol, mmol·l^-1^** | 3.49±0.95 | 3.51±0.95 | 3.46±0.95 |
| **LDL cholesterol, mmol·l^-1^** | 2.79±0.84 | 2.81±0.85 | 2.73±0.83 |
| **Dyslipidaemia, n (%)** | 12,856 (82.1) | 9,175 (82.8) | 3,681 (80.4) |
| **Systolic BP, mmHg** | 138.1±18.0 | 138.4±18.0 | 137.3±18.2 |
| **Diastolic BP, mmHg** | 78.8±9.4 | 79.2±9.5 | 77.6±9.2 |
| **Pulse pressure, mmHg** | 59.3±15.7 | 59.1±15.5 | 59.7±16.1 |
| **Hypertension, n (%)** | 13,096 (83.6) | 9,410 (84.9) | 3,686 (80.5) |
| **Anti-hyperglycaemic treatment, n (%)** |  |  |  |
| **Lifestyle** | 2,113 (13.5) | 1,494 (13.5) | 619 (13.5) |
| **Non-insulin** | 9,619 (61.4) | 6,853 (61.9) | 2,766 (60.4) |
| **Insulin** | 3,924 (25.1) | 2,731 (24.7) | 1,193 (26.1) |
| **Lipid-lowering treatment, n (%)** | 7,238 (46.2) | 5,206 (47.0) | 2,032 (44.4) |
| **Anti-hypertensive treatment, n (%)** | 11,072 (70.7) | 7,935 (71.6) | 3,137 (68.5) |
| **Anti-platelet treatment, n (%)** | 6,248 (39.9) | 4,415 (39.9) | 1,833 (40.0) |
| **Anti-coagulant treatment, n (%)** | 669 (4.3) | 503 (4.5) | 166 (3.6) |
| **Albuminuria, mg·day^-1^** | 72.3±317.0 | 82.3±35.9 | 78.6±30.7 |
| **Serum creatinine, μmol·l^-1^** | 81.1±34.5 | 73.0±341.5 | 70.6±247.8 |
| **eGFR, ml·min^-1^·1.73m^-2^** | 80.3±20.9 | 78.9±20.9 | 83.6±20.7 |
| **DKD phenotype, n (%)** |  |  |  |
| **No DKD** | 9,984 (63.8) | 6,994 (63.1) | 2,990 (65.3) |
| **Albuminuric DKD with preserved eGFR** | 2,966 (18.9) | 2,010 (18.1) | 956 (20.9) |
| **Nonalbuminuric DKD** | 1,476 (9.4) | 1,153 (10.4) | 323 (7.1) |
| **Albuminuric DKD with reduced eGFR** | 1,230 (7.9) | 921 (8.3) | 309 (6.7) |
| **DR, n (%)** |  |  |  |
| **No DR** | 12,189 (77.9) | 8,749 (79.0) | 3,440 (75.1) |
| **Non-advanced DR** | 1,947 (12.4) | 1,369 (12.4) | 578 (12.6) |
| **Advanced DR** | 1,520 (9.7) | 960 (8.7) | 560 (12.2) |
| **CVD, n (%)** |  |  |  |
| **Any** | 3,620 (23.1) | 2,754 (24.9) | 866 (18.9) |
| **Myocardial infarction** | 1,742 (11.1) | 1,250 (11.3) | 492 (10.7) |
| **Coronary revascularization** | 1,579 (10.1) | 1,153 (10.4) | 426 (9.3) |
| **Any coronary event** | 2,396 (15.3) | 1,766 (15.9) | 630 (13.8) |
| **Stroke** | 513 (3.3) | 387 (3.5) | 126 (2.8) |
| **Carotid revascularization** | 856 (5.5) | 780 (7.0) | 76 (1.7) |
| **Any carotid event** | 1,292 (8.3) | 1,107 (10.0) | 185 (4.0) |
| **Ulcer/gangrene/amputation** | 556 (3.6) | 417 (3.8) | 139 (3.0) |
| **Lower limb revascularization** | 450 (2.9) | 372 (3.4) | 78 (1.7) |
| **Any peripheral event** | 883 (5.6) | 695 (6.3) | 188 (4.1) |
| **Comorbidities n (%)** |  |  |  |
| **Any** | 2,787 (17.8) | 1,979 (17.9) | 808 (17.6) |
| **COPD** | 674 (4.3) | 373 (3.4) | 301 (6.6) |
| **Chronic liver disease** | 1,361 (8.7) | 1,051 (9.5) | 310 (6.8) |
| **Cancer** | 1,031 (6.6) | 738 (6.7) | 293 (6.4) |

WC = waist circumference; HbA_1c_ = haemoglobin A_1c_; BMI = body mass index; BP = blood pressure; eGFR = estimated glomerular filtration rate; DKD = diabetic kidney disease; DR = diabetic retinopathy; = CVD = cardiovascular disease; COPD = chronic obstructive pulmonary disease.
