# Supplementary material for: Body mass index versus surrogate measures of central adiposity as independent predictors of mortality in type 2 diabetes
Source: Cardiovasc Diabetol. 2022 Dec 2;21:266. doi: 10.1186/s12933-022-01706-2 (PMC9716975; doi:10.1186/s12933-022-01706-2)
Supplement: Supplementary file 7 — Additional file 7: Table S4. Univariate correlations of surrogate measures of central adiposity between each other and with BMI by Pearson correlation coefficient. [file 12933_2022_1706_MOESM7_ESM.doc]

**Additional file 7: Table S4.** Univariate correlations of surrogate measures of central adiposity between each other and with BMI by Pearson correlation coefficient.

|  | **WC** | | **WHtR** | | **ABSI** | | **BMI** | |
| --- | --- | --- | --- | --- | --- | --- | --- | --- |
|  | ***r*** | ***P*** | ***r*** | ***P*** | ***r*** | ***P*** | ***R*** | ***P*** |
| **WC** | 1.000 | - | 0.894 | <0.0001 | 0.386 | <0.0001 | 0.825 | <0.0001 |
| **WHtR** | 0.894 | <0.0001 | 1.000 | - | 0.374 | <0.0001 | 0.840 | <0.0001 |
| **ABSI** | 0.386 | <0.0001 | 0.374 | <0.0001 | 1.000 | - | -0.135 | <0.0001 |

WC = waist circumference; WHtR = waist-to-height ratio; ABSI = A Body Shape Index; BMI = body mass index.
