# Supplementary material for: Body mass index versus surrogate measures of central adiposity as independent predictors of mortality in type 2 diabetes
Source: Cardiovasc Diabetol. 2022 Dec 2;21:266. doi: 10.1186/s12933-022-01706-2 (PMC9716975; doi:10.1186/s12933-022-01706-2)
Supplement: Supplementary file 8 — Additional file 8: Table S5. Baseline clinical features of study participants by WC, WHtR, and ABSI tertiles. [file 12933_2022_1706_MOESM8_ESM.doc]

**Additional file 8: Table S5.** Baseline clinical features of study participants by WC, WHtR, and ABSI tertiles.

| **Variables** | **WC tertiles** | | | ***P*** | **WHtR tertiles** | | | ***P*** | **ABSI tertiles** | | | ***P*** |
| --- | --- | --- | --- | --- | --- | --- | --- | --- | --- | --- | --- | --- |
|  | **I** | **II** | **III** |  | **I** | **II** | **III** |  | **I** | **II** | **III** |  |
| **N** | 1,526 | 1,527 | 1,525 |  | 1,528 | 1,526 | 1,524 |  | 1,527 | 1,527 | 1,524 |  |
| **Mean±SD** | 90.5±5.9 | 103.0±31 | 117.8±8.3 |  | 0.55±0.04 | 0.63±0.04 | 0.72±0.07 |  | 0.079±0.003 | 0.085±0.001 | 0.091±0.004 |  |
| **(range)** | (67-98) | (98-110) | (107-160) |  | (0.37-0.63) | (0.57-0.71) | (0.63-1.00) |  | (0.061-0.083) | (0.083-0.088) | (0.086-0.116) |  |
| **Deaths, n (%)** | 265 (17.4) | 252 (16.5) | 352 (23.1) | <0.0001 | 240 (15.7) | 253 (16.6) | 376 (24.7) | <0.0001 | 219 (14.3) | 258 (16.9) | 392 (25.7) | <0.0001 |
| **Age, years** | 63.4±10.3 | 64.8±9.3 | 64.0±9.7 | <0.0001 | 62.6±10.3 | 64.5±9.3 | 65.0±9.6 | <0.0001 | 60.9±10.4 | 64.1±9.1 | 67.2±8.9 | <0.0001 |
| **Sex, n (%)** |  |  |  | 1.000 |  |  |  | 0.999 |  |  |  | 1.000 |
| **Females** | 651 (42.7) | 651 (42.6) | 650 (42.6) |  | 652 (42.7) | 651 (42.7) | 649 (42.6) |  | 651 (42.6) | 651 (42.6) | 650 (42.7) |  |
| **Males** | 875 (57.3) | 876 (57.4) | 875 (57.4) |  | 876 (57.3) | 875 (57.3) | 875 (57.4) |  | 876 (57.4) | 876 (57.4) | 874 (57.3) |  |
| **Smoking, n (%)** |  |  |  | <0.0001 |  |  |  | <0.0001 |  |  |  | 0.129 |
| **Never** | 836 (54.8) | 817 (53.5) | 798 (52.3) |  | 827 (54.1) | 806 (52.8) | 818 (53.7) |  | 810 (53.0) | 821 (53.8) | 820 (53.8) |  |
| **Former** | 373 (24.4) | 456 (29.9) | 480 (31.5) |  | 375 (24.5) | 463 (30.3) | 471 (30.9) |  | 426 (27.9) | 422 (27.6) | 461 (30.2) |  |
| **Current** | 317 (20.8) | 254 (16.6) | 247 (16.2) |  | 326 (21.3) | 257 (16.8) | 235 (15.4) |  | 291 (19.1) | 284 (18.6) | 243 (15.9) |  |
| **Physical activity, n (%)** |  |  |  | <0.0001 |  |  |  | <0.0001 |  |  |  | 0.416 |
| **Inactive or moderately inactive** | 893 (58.6) | 970 (63.5) | 1,019 (66.8) |  | 894 (58.5) | 948 (62.1) | 1,040 (68.2) |  | 968 (63.4) | 937 (61.4) | 977 (64.1) |  |
| **Moderately active** | 613 (40.2) | 539 (35.3) | 486 (31.9) |  | 611 (40.0) | 561 (36.8) | 466 (30.6) |  | 536 (35.1) | 571 (37.4) | 531 (34.8) |  |
| **Highly active** | 20 (1.3) | 18 (1.2) | 20 (1. 3) |  | 23 (1.5) | 17 (1.1) | 18 (1.2) |  | 23 (1.5) | 19 (1.2) | 16 (1.0) |  |
| **Diabetes duration, years** | 11.1±8.9 | 10.7±9.0 | 10.9±9.2 | 0.516 | 10.8±8.9 | 10.6±8.8 | 11.3±9.5 | 0.101 | 9.9±8.6 | 10.7±9.0 | 12.1±9.5 | <0.0001 |
| **HbA_1c_, %** | 7.30±1.60 | 7.23±1.42 | 7.67±1.70 | <0.0001 | 7.26±1.58 | 7.27±1.49 | 7.67±1.67 | <0.0001 | 7.26±1.57 | 7.37±1.52 | 7.56±1.66 | <0.0001 |
| **BMI, kg·m^-2^** | 25.2±2.8 | 29.1±3.0 | 34.5±4.8 | <0.0001 | 25.2±2.7 | 29.2±3.1 | 34.4±4.8 | <0.0001 | 30.2±5.7 | 29.8±5.2 | 28.8±4.8 | <0.0001 |
| **WC, cm** | - | - | - | - | 91.3±6.8 | 103.1±5.2 | 116.8±9.3 | <0.0001 | 97.9±12.0 | 104.4±11.8 | 108.9±11.9 | <0.0001 |
| **WHtR, cm·m^-1^** | 0.56±0.04 | 0.63±0.04 | 0.72±0.07 | <0.0001 | - | - | - | - | 0.60±0.08 | 0.64±0.08 | 0.67±0.08 | <0.0001 |
| **ABSI** | 0.083±0.005 | 0.085±0.005 | 0.087±0.005 | <0.0001 | 0.083±0.005 | 0.085±0.005 | 0.087±0.005 | <0.0001 | - | - | - | - |
| **Triglycerides, mmol·l^-1^** | 1.42±1.05 | 1.66±1.13 | 1.79±1.10 | <0.0001 | 1.43±1.05 | 1.67±1.15 | 1.78±1.09 | <0.0001 | 1.58±0.99 | 1.66±1.14 | 1.64±1.17 | 0.119 |
| **Total cholesterol, mmol·l^-1^** | 4.78±1.01 | 4.73±1.02 | 4.72±0.95 | 0.151 | 4.76±0.98 | 4.79±1.04 | 4.68±0.95 | <0.0001 | 4.80±0.96 | 4.77±1.01 | 4.67±1.00 | 0.001 |
| **HDL cholesterol, mmol·l^-1^** | 1.34±0.38 | 1.28±0.36 | 1.24±0.33 | <0.0001 | 1.34±0.39 | 1.28±0.35 | 1.24±0.34 | <0.0001 | 1.28±0.37 | 1.28±0.34 | 1.29±0.37 | 0.656 |
| **Triglycerides:HDL ratio** | 2.79±2.81 | 3.35±2.69 | 3.71±2.88 | <0.0001 | 2.83±2.90 | 3.35±2.66 | 3.67±2.84 | <0.0001 | 3.21±2.59 | 3.32±2.78 | 3.32±3.07 | 0.497 |
| **Non-HDL cholesterol, mmol·l^-1^** | 3.44±0.97 | 3.45±0.97 | 3.48±0.91 | <0.0001 | 3.42±0.95 | 3.51±0.99 | 3.45±0.91 | <0.0001 | 3.51±0.92 | 3.49±0.96 | 3.37±0.96 | <0.0001 |
| **LDL cholesterol, mmol·l^-1^** | 2.80±0.86 | 2.71±0.83 | 2.68±0.80 | 0.487 | 2.78±0.84 | 2.76±0.85 | 2.66±0.80 | <0.0001 | 2.81±0.83 | 2.75±0.84 | 2.64±0.82 | <0.0001 |
| **Dyslipidaemia, n (%)** | 1,210 (79.3) | 1,222 (80.0) | 1,249 (81.9) | 0.173 | 1,204 (78.8) | 1,239 (81.2) | 1,238 (81.2) | 0.151 | 1,247 (81.7) | 1,231 (80.6) | 1,203 (78.9) | 0.160 |
| **Systolic BP, mmHg** | 134.3±17.1 | 138.2±18.5 | 139.5±18.4 | <0.0001 | 134.2±17.2 | 138.3±18.4 | 139.5±18.4 | <0.0001 | 134.3±16.9 | 138.4±17.8 | 139.2±19.4 | <0.0001 |
| **Diastolic BP, mmHg** | 76.5±8.8 | 77.7±9.3 | 78.6±9.5 | <0.0001 | 76.7±8.7 | 78.1±9.3 | 78.1±9.6 | <0.0001 | 78.2±8.9 | 78.2±9.3 | 76.5±9.4 | <0.0001 |
| **Pulse pressure, mmHg** | 57.7±15.2 | 60.4±16.5 | 60.9±16.3 | <0.0001 | 57.5±15.5 | 60.2±16.0 | 61.4±16.5 | <0.0001 | 56.1±14.6 | 60.2±15.4 | 62.7±17.4 | <0.0001 |
| **Hypertension, n (%)** | 1,101 (72.1) | 1,242 (81.3) | 1,343 (88.1) | <0.0001 | 1,079 (70.6) | 1,259 (82.5) | 1,348 (88.5) | <0.0001 | 1,149 (75.2) | 1,257 (82.3) | 1,280 (84.0) | <0.0001 |
| **Anti-hyperglycaemic treatment, n (%)** |  |  |  | <0.0001 |  |  |  | <0.0001 |  |  |  | 0.001 |
| **Lifestyle** | 258 (16.9) | 214 (14.0) | 147 (9.6) |  | 264 (17.3) | 204 (13.4) | 151 (9.9) |  | 229 (15.0) | 208 (13.6) | 182 (11.9) |  |
| **Non-insulin** | 892 (58.5) | 960 (62.9) | 914 (59.9) |  | 887 (58.0) | 984 (64.5) | 895 (58.7) |  | 938 (61.4) | 939 (61.5) | 889 (58.3) |  |
| **Insulin** | 376 (24.6) | 353 (23.1) | 464 (30.4) |  | 377 (24.7) | 338 (22.1) | 478 (31.4) |  | 360 (23.6) | 380 (24.9) | 453 (29.7) |  |
| **Lipid-lowering treatment, n (%)** | 640 (41.9) | 693 (45.4) | 699 (45.8) | 0.060 | 612 (40.1) | 726 (47.6) | 694 (45.5) | <0.0001 | 656 (43.0) | 665 (43.5) | 711 (46.7) | 0.088 |
| **Anti-hypertensive treatment, n (%)** | 884 (57.9) | 1,061 (69.5) | 1,192 (78.2) | <0.0001 | 867 (56.7) | 1,078 (70.6) | 1,192 (78.2) | <0.0001 | 993 (65.0) | 1,046 (68.5) | 1,098 (72.0) | <0.0001 |
| **Anti-platelet treatment, n (%)** | 552 (36.2) | 626 (41.0) | 655 (43.0) | <0.0001 | 538 (35.2) | 623 (40.8) | 672 (44.1) | <0.0001 | 571 (37.4) | 610 (39.9) | 652 (42.8) | 0.010 |
| **Anti-coagulant treatment, n (%)** | 48 (3.1) | 47 (3.1) | 71 (4.7) | 0.031 | 52 (3.4) | 44 (2.9) | 70 (4.6) | 0.035 | 48 (3.1) | 50 (3.3) | 68 (4.5) | 0.100 |
| **Albuminuria, mg·day^-1^** | 53.1±219.7 | 55.6±167.2 | 103.1±326.3 | <0.0001 | 51.8±207.2 | 58.4±207.3 | 101.7±311.3 | <0.0001 | 65.7±221.7 | 63.7±225.8 | 82.5±289.7 | 0.071 |
| **Serum creatinine, μmol·l^-1^** | 77.0±34.3 | 77.9±26.2 | 80.6±30.8 | 0.004 | 76.8±31.9 | 77.5±27.8 | 81.2±31.8 | <0.0001 | 77.8±32.5 | 77.4±28.8 | 80.3±30.4 | 0.016 |
| **eGFR, ml·min^-1^·1.73m^-2^** | 85.6±20.0 | 83.0±19.7 | 82.3±22.1 | <0.0001 | 85.9±19.7 | 83.6±19.6 | 81.3±22.4 | <0.0001 | 86.2±20.9 | 84.3±19.6 | 80.3±21.0 | <0.0001 |
| **DKD phenotype, n (%)** |  |  |  | <0.0001 |  |  |  | <0.0001 |  |  |  | <0.0001 |
| **No DKD** | 1,092 (71.6) | 1,014 (66.4) | 884 (58.0) |  | 1,110 (72.6) | 1,021 (66.9) | 859 (56.4) |  | 1,025 (67.1) | 1,031 (67.5) | 934 (61.3) |  |
| **Albuminuric DKD with preserved eGFR** | 258 (16.9) | 304 (19.9) | 394 (25.8) |  | 252 (16.5) | 308 (20.2) | 396 (26.0) |  | 317 (20.8) | 314 (20.6) | 325 (21.3) |  |
| **Nonalbuminuric DKD** | 93 (6.1) | 114 (7.5) | 116 (7.6) |  | 86 (5.6) | 108 (7.1) | 129 (8.5) |  | 83 (5.4) | 107 (7.0) | 133 (8.7) |  |
| **Albuminuric DKD with reduced eGFR** | 83 (5.4) | 95 (6.2) | 131 (8.6) |  | 80 (5.2) | 89 (5.8) | 140 (9.2) |  | 102 (6.7) | 75 (4.9) | 132 (8.7) |  |
| **DR, n (%)** |  |  |  | <0.0001 |  |  |  | <0.0001 |  |  |  | 0.015 |
| **No DR** | 1,181 (77.4) | 1,179 (77.2) | 1,080 (70.8) |  | 1,189 (77.8) | 1,167 (76.5) | 1,084 (71.1) |  | 1,181 (77.3) | 1,160 (76.0) | 1,099 (72.1) |  |
| **Non-advanced DR** | 181 (11.9) | 179 (11.7) | 218 (14.3) |  | 186 (12.2) | 183 (12.0) | 209 (13.7) |  | 171 (11.2) | 188 (12.3) | 219 (14.4) |  |
| **Advanced DR** | 164 (10.7) | 169 (11.1) | 227 (14.9) |  | 153 (10.0) | 176 (11.5) | 231 (15.2) |  | 175 (11.5) | 179 (11.7) | 206 (13.5) |  |
| **CVD, n (%)** |  |  |  |  |  |  |  |  |  |  |  |  |
| **Any** | 253 (16.6) | 286 (18.7) | 327 (21.4) | 0.003 | 247 (16.2) | 280 (18.3) | 339 (22.2) | <0.0001 | 243 (15.9) | 274 (17.9) | 349 (22.9) | <0.0001 |
| **Myocardial infarction** | 147 (9.6) | 160 (10.5) | 185 (12.1) | 0.077 | 138 (9.0) | 166 (10.9) | 188 (12.3) | 0.013 | 136 (8.9) | 155 (10.2) | 201 (13.2) | <0.0001 |
| **Coronary revascularization** | 136 (8.9) | 143 (9.4) | 147 (9.6) | 0.784 | 130 (8.5) | 142 (9.3) | 154 (10.1) | 0.316 | 132 (8.6) | 134 (8.8) | 160 (10.5) | 0.144 |
| **Any coronary event** | 191 (12.5) | 209 (13.7) | 230 (15.1) | 0.120 | 184 (12.0) | 211 (13.8) | 235 (15.4) | 0.025 | 174 (11.4) | 204 (13.4) | 252 (16.5) | <0.0001 |
| **Stroke** | 37 (2.4) | 38 (2.5) | 51 (3.3) | 0.223 | 30 (2.0) | 43 (2.8) | 53 (3.5) | 0.037 | 35 (2.3) | 41 (2.7) | 50 (3.3) | 0.244 |
| **Carotid revascularization** | 24 (1.6) | 23 (1.5) | 29 (1.9) | 0.658 | 24 (1.6) | 21 (1.4) | 31 (2.0) | 0.344 | 24 (1.6) | 24 (1.6) | 28 (1.8) | 0.803 |
| **Any carotid event** | 57 (3.7) | 54 (3.5) | 74 (4.9) | 0.138 | 52 (3.4) | 58 (3.8) | 75 (4.9) | 0.087 | 54 (3.5) | 58 (3.8) | 73 (4.8) | 0.179 |
| **Ulcer/gangrene/amputation** | 38 (2.5) | 36 (2.4) | 65 (4.3) | 0.003 | 39 (2.6) | 29 (1.9) | 71 (4.7) | <0.0001 | 34 (2.2) | 38 (2.5) | 67 (4.4) | 0.001 |
| **Lower limb revascularization** | 16 (1.0) | 29 (1.9) | 33 (2.2) | 0.045 | 18 (1.2) | 26 (1.7) | 34 (2.2) | 0.080 | 17 (1.1) | 18 (1.2) | 43 (2.8) | <0.0001 |
| **Any peripheral event** | 48 (3.1) | 54 (3.5) | 86 (5.6) | 0.001 | 51 (3.3) | 46 (3.0) | 91 (6.0) | <0.0001 | 47 (3.1) | 52 (3.4) | 89 (5.8) | <0.0001 |
| **Comorbidities n (%)** |  |  |  |  |  |  |  |  |  |  |  |  |
| **Any** | 236 (15.5) | 282 (18.5) | 290 (19.0) | 0.022 | 227 (14.9) | 274 (18.0) | 307 (20.1) | 0.001 | 251 (16.4) | 227 (14.9) | 330 (21.7) | <0.0001 |
| **COPD** | 72 (4.7) | 101 (6.6) | 128 (8.4) | <0.0001 | 63 (4.1) | 101 (6.6) | 137 (9.0) | <0.0001 | 87 (5.7) | 76 (5.0) | 138 (9.1) | <0.0001 |
| **Chronic liver disease** | 97 (6.4) | 112 (7.3) | 101 (6.6) | 0.539 | 93 (6.1) | 106 (6.9) | 111 (7.3) | 0.398 | 92 (6.0) | 90 (5.9) | 128 (8.4) | 0.008 |
| **Cancer** | 94 (6.2) | 100 (6.5) | 99 (6.5) | 0.894 | 94 (6.2) | 100 (6.6) | 99 (6.5) | 0.887 | 98 (6.4) | 86 (5.6) | 109 (7.2) | 0.229 |

WC = waist circumference; WHtR = waist-to-height ratio; ABSI = A Body Shape Index; HbA_1c_ = haemoglobin A_1c_; BMI = body mass index; BP = blood pressure; eGFR = estimated glomerular filtration rate; DKD = diabetic kidney disease; DR = diabetic retinopathy; = CVD = cardiovascular disease; COPD = chronic obstructive pulmonary disease.
